# TRAP5 Inhibition Targeting Scar‐Associated Macrophages Ameliorates Acute Kidney Injury to Chronic Kidney Disease Transition

**DOI:** 10.1002/advs.202519855

**Published:** 2026-03-03

**Authors:** Chenxi Wang, Yaodong Gu, Wen Du, Lin Xie, Jinwei Quan, Yu Zhao, Ye Cheng, Zhaonan Wei, Yuanyuan Sha, Yi Wang, Dechao Xu, Xiang Gao, Min Chen, Xiangchen Gu

**Affiliations:** ^1^ Department of Nephrology Yueyang Hospital of Integrated Traditional Chinese and Western Medicine Shanghai University of Traditional Chinese Medicine Shanghai China; ^2^ Department of Nephrology Ruijin Hospital Luwan Branch Shanghai Jiaotong University School of Medicine Shanghai China; ^3^ Department of Nephrology Shanghai Fourth People's Hospital Tongji University Shanghai China; ^4^ Department of Nephrology Ningbo Yinzhou No.2 Hospital Ningbo China; ^5^ Department of Nephrology The Second Affiliated Hospital of Soochow University Suzhou China; ^6^ Department of Intensive Care Unit Yueyang Hospital of Integrated Traditional Chinese and Western Medicine Shanghai University of Traditional Chinese Medicine Shanghai China; ^7^ Department of Nephrology Changzheng Hospital Naval Medical University Shanghai China; ^8^ Department of Medicine Shanghai Hospital of Civil Aviation Administration of China Shanghai China

**Keywords:** ACP5/TRAP5, AKI to CKD transition, Aristolochic Acid I, Macrophage, single‐cell RNA sequencing

## Abstract

The transition from acute kidney injury (AKI) to chronic kidney disease (CKD) lacks effective therapies. Using a murine aristolochic acid I model (AAI), followed by a 2 week remodeling phase, we mapped macrophage states across the AKI to CKD transition. Single‐cell RNA sequencing of kidneys from control, acute, and remodeling stages profiled 39 345 cells spanning 18 clusters. Macrophage subclustering and trajectories revealed emergence of scar‐associated macrophages (SMs) marked by high Acp5 (encoding tartrate‐resistant acid phosphatase 5 (TRAP5)) and enriched during the remodeling phase. Cell–cell communication highlighted a dominant Spp1–Cd44 axis driving SM activation. Immunofluorescence confirmed TRAP5^+^CD68^+^ macrophage accumulation in multiple murine CKD and human CKD biopsies.

Functionally, pharmacological TRAP5 inhibition conferred robust protection against AKI–CKD transition. TRAP5 blockade initiated in the AKI phase markedly attenuated fibrosis, inflammation, and renal dysfunction in both AAI and ischemia‐reperfusion injury (IRI) models; while preinhibition similarly mitigated unilateral ureteral obstruction (UUO)‐induced fibrosis. In bone marrow‐derived macrophages (BMDMs), TRAP5 blockade abrogated osteopontin (Spp1)‐driven metabolic and profibrotic reprogramming.

In conclusion, TRAP5^+^ scar‐associated macrophages are disease‐promoting effectors of maladaptive remodeling in AKI–CKD transition. Targeting TRAP5 not only suppresses profibrotic macrophage activation but also protects renal structure and function across multiple models, establishing TRAP5 inhibition as a promising therapeutic strategy to halt CKD progression.

## Graphical abstract

This study delineates macrophage heterogeneity along the acute kidney injury to chronic kidney disease transition. Single‐cell RNA sequencing reveals a TRAP5^+^ scar‐associated macrophage subset driven by *Spp1*–*C* *d* *44* signaling and mitochondrial metabolic reprogramming. Pharmacologic TRAP5 inhibition across multiple injury models attenuates profibrotic macrophage activation, mitigates fibrosis and inflammation, and preserves renal function, positioning TRAP5 as a promising therapeutic target.

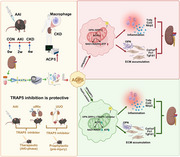

## 1 Introduction

Acute kidney injury (AKI) is a sudden loss of renal function, leading to the accumulation of metabolic waste, water, and electrolytes [1]. AKI significantly increases the risk of progression to chronic kidney disease (CKD), a state of irreversible renal damage characterized by fibrosis and declining kidney function, and ultimately to end‐stage renal disease (ESRD). Thus, the AKI to CKD transition represents a critical challenge in nephrology, contributing substantially to healthcare burden and patient morbidity.

Recent studies have identified multiple intrinsic mechanisms contributing to the AKI to CKD transition, including mitochondrial dysfunction [2], metabolic reprogramming [3], and ferroptosis [4] mediated renal tubular injury. Alongside these intrinsic pathways, immune‐mediated mechanisms, particularly involving macrophages, play essential roles in modulating renal inflammation, tissue repair, and fibrosis [5, 6]. During AKI, proinflammatory macrophages release cytokines and chemokines that exacerbate renal inflammation. Persistent activation of these macrophages can lead to chronic inflammation, resulting in tissue scarring and contributing to CKD development [7]. Despite these advances, the complex and coordinated interactions among macrophages and renal resident cells during AKI to CKD progression remain incompletely defined.

Single‐cell RNA sequencing (scRNA‐seq) has profoundly advanced our understanding of cellular dynamics by enabling comprehensive profiling of heterogenous cell populations and their functional states [8, 9, 10]. Previous scRNA‐seq studies have indicated sustained populations of Mincle‐high neutrophils and macrophages as key drivers of chronic inflammation and fibrosis through TNF‐α signaling pathways [11]. More recently, distinct macrophage subsets such as CD38hi macrophages have been implicated in mediating NAD^+^ metabolism, promoting tubular cell senescence and renal fibrosis, suggesting metabolic modulation of macrophages as a therapeutic strategy [12]. Nevertheless, most mechanistic insights arise from studies using ischemia‐reperfusion injury (IRI) models. Although IRI can trigger AKI and drive AKI to CKD transition, its reperfusion‐driven oxidative and inflammatory injury captures only a part of etiologic spectrum underlying progressive human kidney remodeling postinjury. Moreover, several studies performed scRNA‐seq on macrophages presorted by flow cytometry. While this approach enhances myeloid resolution, it limits our assessment on cross‐talk between macrophages and other immune and resident renal cells. Finally, despite revealing novel subclusters and candidate signaling axes, in vivo‐validated therapeutic targets remain scarce. These gaps motivate broader models and integrative single‐cell analyses to resolve multicellular interactions and identify druggable pathways.

To address these gaps, we applied scRNA‐seq to a well‐established murine model of AKI to CKD transition induced by aristolochic acid I (AAI). In this model, repeated AAI exposures induce recurrent tubular injury, and subsequent AAI withdrawal initiates partial recovery followed by fibrotic remodeling and the development of CKD [13, 14, 15]. Using scRNA‐seq, our study specifically aims to identify critical macrophage subpopulations and elucidate their dynamic interactions throughout disease progression, thereby revealing novel therapeutic targets with the potential to mitigate CKD progression.

## 2 Results

### 2.1 Cellular Landscape Reveals Dynamics during the Transition from AKI to CKD

**FIGURE 1:**
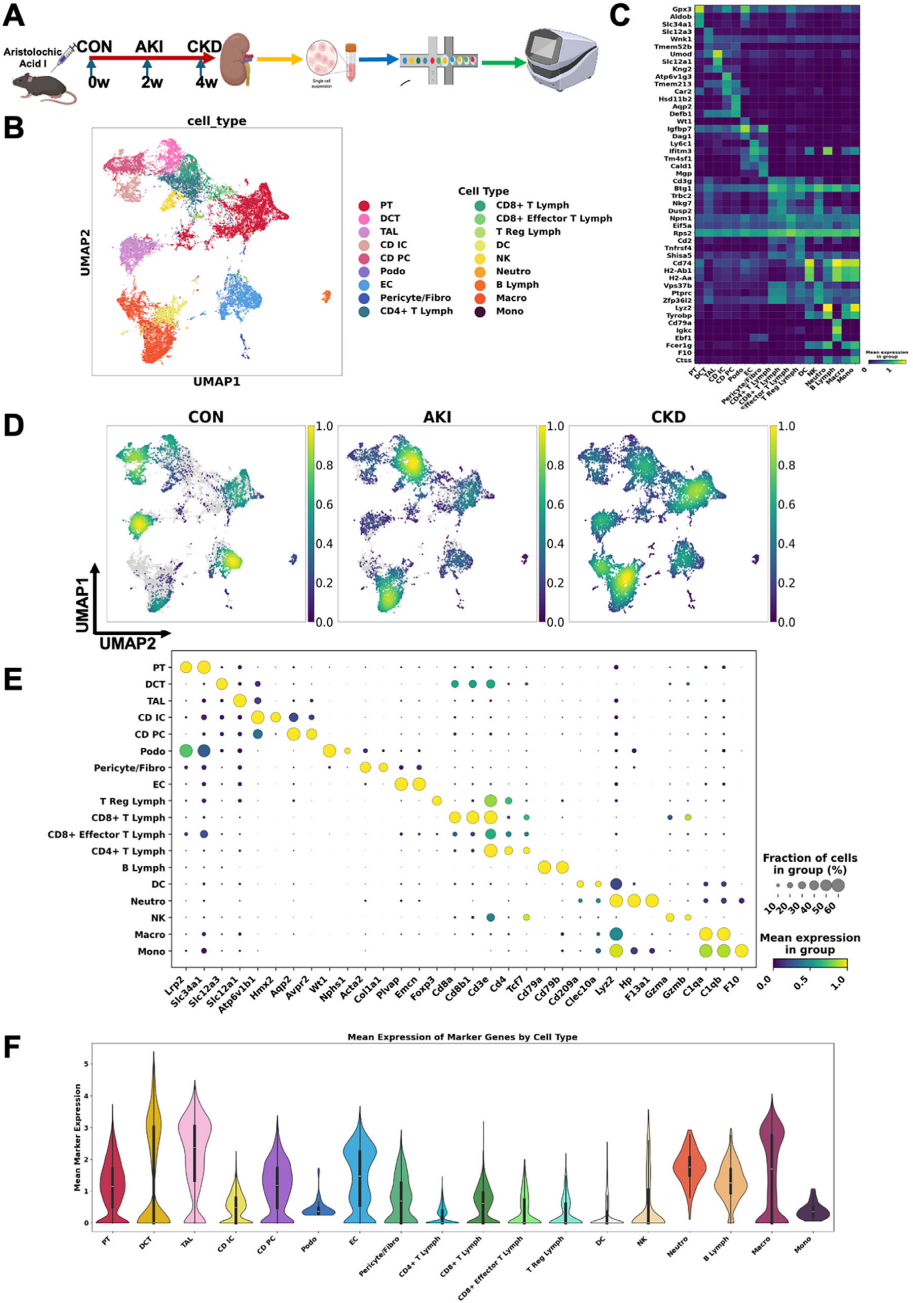
Single‐cell atlas of the kidney across AAI‐induced AKI to CKD transition. (A) Schematic overview of experimental design showing the timeline of Aristolochic Acid I (AAI) administration in mice. Mice were sacrificed at control (CON), acute kidney injury (AKI, 2 weeks), and chronic kidney disease (CKD, 4 weeks) time points for single‐cell transcriptome analysis. (B) UMAP visualization of all sequenced kidney cells, showing cell‐type heterogeneity, including macrophages, T cells, B cells, tubular epithelial cells, and fibroblasts, colored by cell identity. (C) Heatmap of mean normalized expression for selected marker genes across annotated kidney cell populations from scRNA‐seq. Columns denote cell types/subsets (e.g., PT, TAL/LOH, DCT, CD‐PC/IC, podocytes, endothelium, fibroblasts, macrophages, B cells, T/NK), rows are genes; color scale shows low (purple) to high (yellow) expression. (D) UMAP density plots for CON, AKI, and CKD, showing changes in cell density and microenvironmental composition as injury progresses. (E) Bubble plots illustrate key marker gene expression across kidney cell subpopulations. Dot size indicates the fraction of cells expressing the gene; color represents mean expression, highlighting cell identity and activation state. (F) Violin plots show the per‐cell mean expression of curated marker‐gene sets (Methods) for each annotated population.

To investigate the cellular dynamics during the transition from AKI to CKD, we established a murine model of AAI‐induced nephropathy. As depicted in Figure 1A, mice received AAI injection (3 mg/kg) and kidney tissues were harvested at 3 time points: baseline (control, CON), 2 weeks post‐AAI injection (AKI phase), and 4 weeks after initial injection (remodeling phase, following 2 weeks of AAI withdrawal). Single‐cell RNA sequencing was performed using the 10 × Genomics platform.

Following rigorous quality control, we analyzed 39 345 individual cells from kidneys of wild‐type mice subjected into AAI injection. The data was normalized, clustered, and subjected to dimensionality reduction via Uniform Manifold Approximation and Projection (UMAP), identifying 18 unique cell‐type clusters (Figure 1B). These clusters were annotated based on canonical marker gene expression [16], encompassing all major renal resident and immune cell populations, including proximal tubular cells (PT), distal convoluted tubule cells (DCT), thick ascending limb cells (TAL), collecting duct intercalated cells (CD IC), collecting duct principal cells (CD PC), podocytes (Podo), pericytes/fibroblasts (Pericyte/Fibro), endothelial cells (EC), and multiple immune subtypes: CD4^+^ T lymphocytes, CD8^+^ T lymphocytes, T regulatory cells (Treg), dendritic cells (DC), natural killer cells (NK), neutrophils (Neutro), B lymphocytes, macrophages (Macro), and monocytes (Mono) (Figure 1B). The assignment of these clusters was guided by canonical marker gene expression, as demonstrated in the heatmap (Figure 1C), which confirms the specificity of cell type annotation across all cell populations.

To visualize alterations in cellular composition across disease states, UMAP for the control, AKI, and CKD groups were compared (Figure 1D). These plots illustrate pronounced dynamic changes, notably a progressive increase in the proportion of immune cells—particularly macrophages—as kidney injury advanced from AKI to CKD. Essential cell type‐specific markers enabled precise cell identification and annotation across control, AKI, and CKD samples, as demonstrated by the bubble plot (Figure 1E), highlighting the distinct expression patterns across renal and immune compartments (e.g., Lrp2 and Slc34a1 for proximal tubular cells, Slc12a3 for distal convoluted tubules, and C1qa/C1qb for macrophages). The specificity and heterogeneity of marker gene expression within each population are further illustrated by violin plots (Figure 1F).

Together, these analyses reveal a dynamic shift in renal cellular composition during disease progression, characterized by a notable expansion of macrophage populations from AKI to CKD, providing a comprehensive single‐cell atlas of the AKI to CKD transition.

### 2.2 Subclustering Identifies Four Macrophage Subsets Across AKI to CKD Transition

**FIGURE 2:**
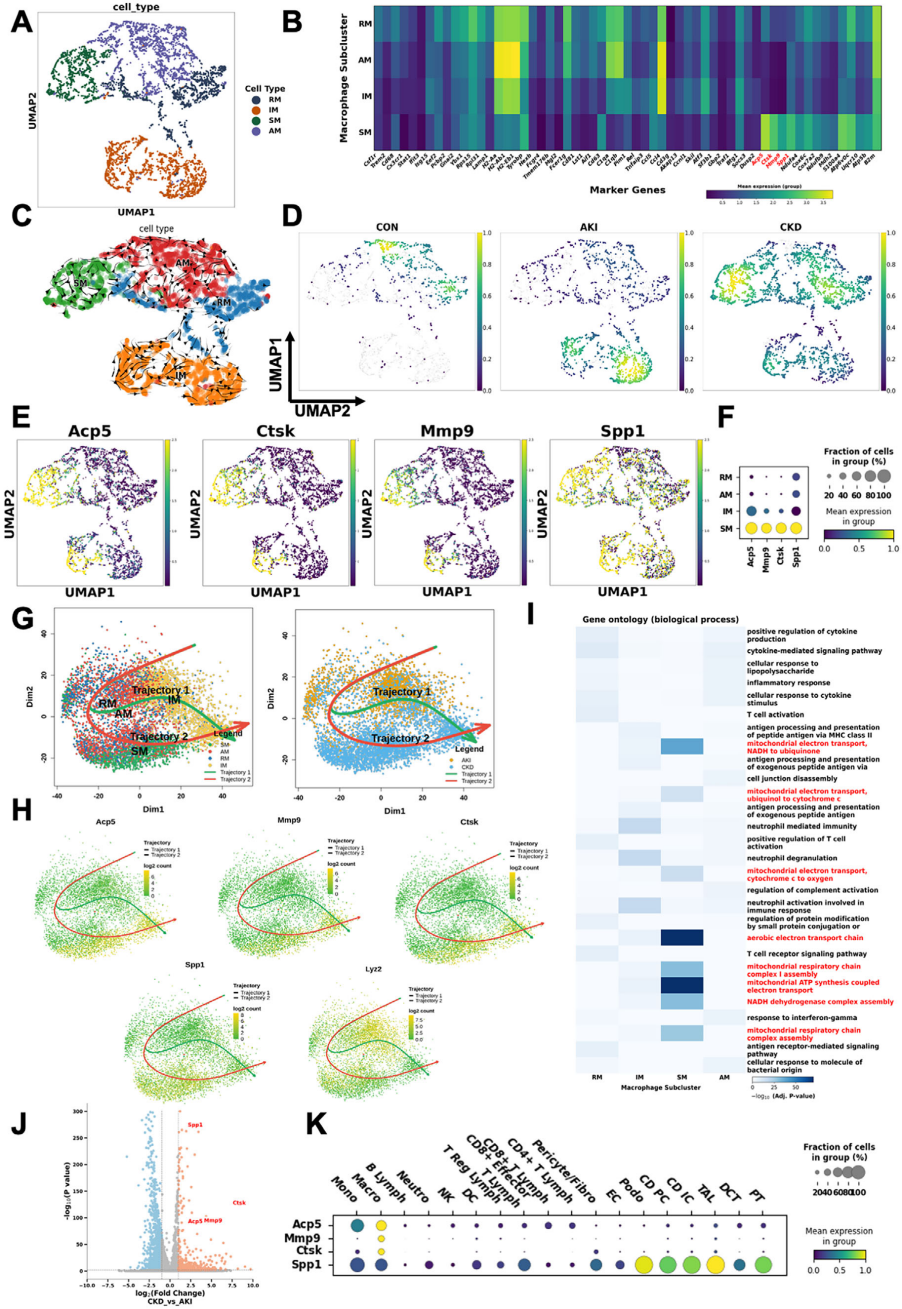
Macrophage subclusters and trajectories during AKI to CKD transition. (A) UMAP projection of kidney macrophages, identifying four transcriptionally distinct subclusters: regulatory macrophages (RM), inflammatory macrophages (IM), scar‐associated macrophages (SM), and antigen‐presenting macrophages (AM). (B) Heatmap of gene expression across macrophage subclusters. Rows list marker genes; column are subclusters(RM, AM, IM, SM). Color within each column encodes the mean normalized expression of that genes among all cells in that subcluster(color bar: purple = low, yellow = high). (C) RNA‐velocity trajectories on the myeloid UMAP. Each dot is a single cell; colors mark subclusters(AM red, RM blue, IM orange, SM green). Black arrows show the projected RNA‐velocity vector field computed from spliced/unspliced counts, indicating each cell's predicted future transcriptional state. Arrow direction denotes the inferred trajectory. (D) Quantitative comparison of macrophage subset frequencies (RM, IM, SM, AM) across CON, AKI, and CKD phases. CON are enriched mainly in regulatory macrophage (RM) territories; AKI shows focal enrichment within inflammatory (IM) regions; CKD exhibits broad enrichment in scar‐associated (SM) territories. (E) UMAP feature plots showing the distribution of macrophage markers (*Acp5*, *Ctsk*, *Mmp9*, *Spp1*). Each point represents one myeloid cell in the UMAP. Color denotes log‐normalized expression for indicated genes. (F) Bubble plot showing the expression patterns of *Acp5*, *Mmp9*, *Ctsk*, and *Spp1* among four macrophage subclusters (RM, AM, IM, and SM). The size of each bubble represents the fraction of cells within the subcluster expressing the gene, while the color intensity reflects the mean expression level (green = low, yellow = high). Notably, *Acp5*, *Mmp9*, *Ctsk*, and *Spp1* are predominantly enriched in scar‐associated macrophages (SM), consistent with their role in extracellular matrix remodeling and fibrotic progression. (G) Slingshot trajectories on UMAP reveal convergence to an IM hub after acute injury with predominant maturation toward an SM fate during remodeling. Trajectory 1 was composed of cells in clusters AM, RM, and IM. Trajectory 2 was composed of AM, RM, and SM. (H) Gene‐level trajectory of *Acp5*, *Ctsk*, *Mmp9*, *Spp1*, and *Lyz2*. (I) Gene Ontology enrichment across macrophage subclusters. Heatmap of GO biological processes enriched in differentially expressed genes from each subcluster (RM, IM, SM, AM). Rows list top terms; Intensity indicates enrichment significance (color scale indicates −log_10_ FDR‐adjusted *p* value). Terms related to mitochondrial respiration/electron transport are highlighted in red to emphasize a coordinated metabolic module. (J) Volcano plot of genes tested for differential expression (CKD vs. AKI); Volcano plot of differential gene expression (CKD vs. AKI) in macrophages. Each point is a gene. The *x*‐axis shows log_2_ fold change (CKD vs. AKI). The *y*‐axis shows −log_10_ (*p* value) from the differential test. Points are colored by outcome: red represents significantly upregulated in CKD, blue represents significantly up in AKI, gray = not significant. Fibrosis/ECM‐remodeling genes *Spp1*, *Ctsk*, *Acp5*, and *Mmp9* are among the most significantly CKD‐enriched. (K) Bubble plot showing the expression patterns of *Acp5*, *Mmp9*, *Ctsk*, and *Spp1* across major renal cell types. The size of each bubble represents the fraction of cells within a cluster expressing the indicated gene, while the color intensity reflects the mean expression level (green = low, yellow = high). Expression of matrix‐remodeling enzymes (*Acp5*, *Mmp9*, and *Ctsk*) was predominantly enriched in macrophages compared to other cell populations, whereas *Spp1* was broadly expressed, with strong signals in both macrophages and renal tubular epithelial cells.

To analyze macrophage heterogeneity, we performed subclustering analysis of macrophages from control, AKI, and CKD phases. This analysis identified four transcriptionally distinct macrophage subpopulations (Figure 2A). Differential gene expression analysis across these subclusters, highlighted their distinct molecular signatures, as visualized in the heatmap (Figure 2B).

One macrophage subcluster was defined by high expression of genes including *Csf1r*, *Trem2*, *Cd68*, and *Cx3cr1*, characteristic markers of regulatory or tissue‐resident macrophages (Figure 2B). The transcriptional profile of this subpopulation suggests functions primarily related to immune homeostasis, tissue maintenance, and phagocytic clearance rather than inflammation. A second subcluster exhibited elevated expression of *H2‐Aa* and *H2‐Eb1* (Figure 2B), encoding major histocompatibility complex class II (MHCII) components involved in antigen presentation, and was accordingly designated as antigen‐presenting macrophages (AM).

In contrast, a third macrophage subcluster prominently expressed inflammatory genes including *Pim1*, *Rel*, *Ccl5*, and *Ccl4*, indicative of classical proinflammatory macrophage activation (Figure 2B). Thus, these cells were classified as inflammatory macrophages (IM). The fourth identified subcluster was distinguished by enriched expression of matrix‐degrading enzymes *Acp5*, *Ctsk*, and *Mmp9*, along with the profibrotic mediator *Spp1*(Figure 2B). The combined expression of these matrix remodeling enzymes and profibrotic cytokine supports a specialized in extracellular matrix turnover and scar‐associated tissue remodeling. Therefore, we designated this subpopulation as “scar‐associated macrophages” (SMs).

RNA velocity analysis further provided insights into the dynamic relationships among these subpopulations, revealing a predominant directional transition from the RM and AM toward the IM and SM clusters, indicative of a macrophage phenotypic shift from homeostatic phenotypes and antigen‐presenting states toward inflammatory and scar‐promoting phenotypes during disease progression (Figure 2C).

Together, these findings underscore the dynamic specialization and plasticity of macrophage subpopulations in response to kidney injury, elucidating their unique roles in immune regulation, antigen presentation, inflammation, and fibrotic tissue remodeling throughout the AKI to CKD transition.

### 2.3 Trajectory and Functional Analyses Uncover ACP5^+^ Scar‐Associated Macrophages as Mediators of Mitochondrial Reprogramming and Fibrosis

Building upon the identification of four transcriptionally distinct macrophage subpopulations (RM, IM, AM, and SM), we next analyzed their distribution patterns across disease stages to characterize dynamic shifts in macrophage phenotypes during the progression from AKI to CKD. As illustrated in Figure 2D, RM and AM subclusters were predominantly enriched in the control group, aligning with their known roles in maintaining immune homeostasis under physiological conditions. In contrast, IM exhibited substantial expansion during the AKI phase, consistent with their role in driving acute inflammatory responses. Remarkably, SM became the predominant macrophage population during the CKD phase, emphasizing their critical involvement in fibrotic tissue remodeling.

Further supporting these findings, UMAP visualization of key matrix‐degrading enzymes (*Acp5*, *Ctsk*, and *Mmp9*) and profibrotic mediator *Spp1* confirmed their selective enrichment within the SM subpopulation (Figure 2E,F). To delineate the dynamic transitions of renal macrophages during disease progression, we performed pseudotime trajectory analysis using both Slingshot and Monocle analyses (Figure 2G; Figure S1A). Slingshot analysis (Figure 2G) showed that following acute injury, macrophages initially transitioned from RM and AM toward the IM phenotype, indicating an early inflammatory response during AKI. As injury progressed and remodeling ensued, macrophage differentiation predominantly shifted toward the SM fate. Along the SM‐directed trajectory, *Acp5*, *Ctsk*, *Mmp9*, and *Spp1* increased progressively (Figure 2H). Monocle analysis (Figure S1A) provided complementary insights, showing a clear pseudotime continuum along which macrophages transitioned from RM/AM to IM and subsequently to SM. Notably, the transition toward the SM fate was accompanied by a progressive upregulation of matrix‐degrading and profibrotic genes‐including *Acp5*, *Ctsk*, *Mmp9*, and *Spp1*– as visualized along the pseudotime trajectory (Figure S1B).

To systematically explore the functional specializations among macrophage subsets, we performed gene ontology (GO) enrichment analysis on differentially expressed genes (DEGs) across RM, AM, IM, and SM clusters. The GO heatmap (Figure 2I) indicated that SM subpopulation uniquely enriched pathways involved in mitochondrial metabolism and electron transport, including “aerobic electron transport chain”, “mitochondrial ATP synthesis coupled electron transport”, and “mitochondrial electron transport, NADH to ubiquinone”. Conversely, IM macrophages were primarily enriched for pathways related to neutrophil‐mediated immunity and antigen processing, while RM and AM subclusters were enriched for cytokine‐mediated signaling, T cell activation and inflammatory response pathways. Thus, each macrophage subcluster clearly adopts distinct function in response to renal injury, with SM notably displaying pronounced metabolic reprogramming.

To further understand genes specifically associated with fibrotic progression, we performed differential expression analysis comparing CKD versus AKI macrophage subpopulations (Figure 2J). The resulting volcano plot demonstrated significant upregulation of matrix‐remodeling and profibrotic genes (notably *Spp1*, *Acp5*, *Ctsk*, and *Mmp9*) in CKD macrophages. Subsequent GO enrichment analysis further clarified the distinct functional adaptations across disease stages. In AKI, macrophages prominently exhibited enrichment for inflammatory and immune‐related processes including cytokine signaling, and neutrophil‐mediated immunity, consistent with their acute inflammatory role. In contrast, CKD macrophages displayed strong enrichment of mitochondrial metabolism pathways, including electron transport and ATP synthesis (Figure S2A). Consistent with these findings, the bubble plot and UMAP feature plot analysis further illustrated that *Acp5*, together with the matrix‐remodeling enzymes *Mmp9* and *Ctsk*, is highly enriched in macrophages compared with other cell types (Figure 2K; Figure S2B,C). Notably, *Acp5* expression was concentrated in CKD‐phase macrophages, highlighting that macrophages are the predominant source of TRAP5 and underscoring their pivotal role in driving extracellular matrix remodeling during disease progression.

Together, these analyses revealed phased rewiring of macrophage phenotypes throughout kidney injury progression. AKI features an IM‐dominated inflammatory response, whereas CKD is driven by SMs specialized for metabolic reprogramming, sustained extracellular‐matrix remodeling. Notably, *Acp5* ^+^ SM emerge as key effectors linking acute injury to chronic scarring, indicating *Acp5* as both a mechanistic marker and a therapeutic target.

### 2.4 Cell to Cell Interaction Analysis Reveals SM‐Centered Signaling Networks during CKD Phase

**FIGURE 3:**
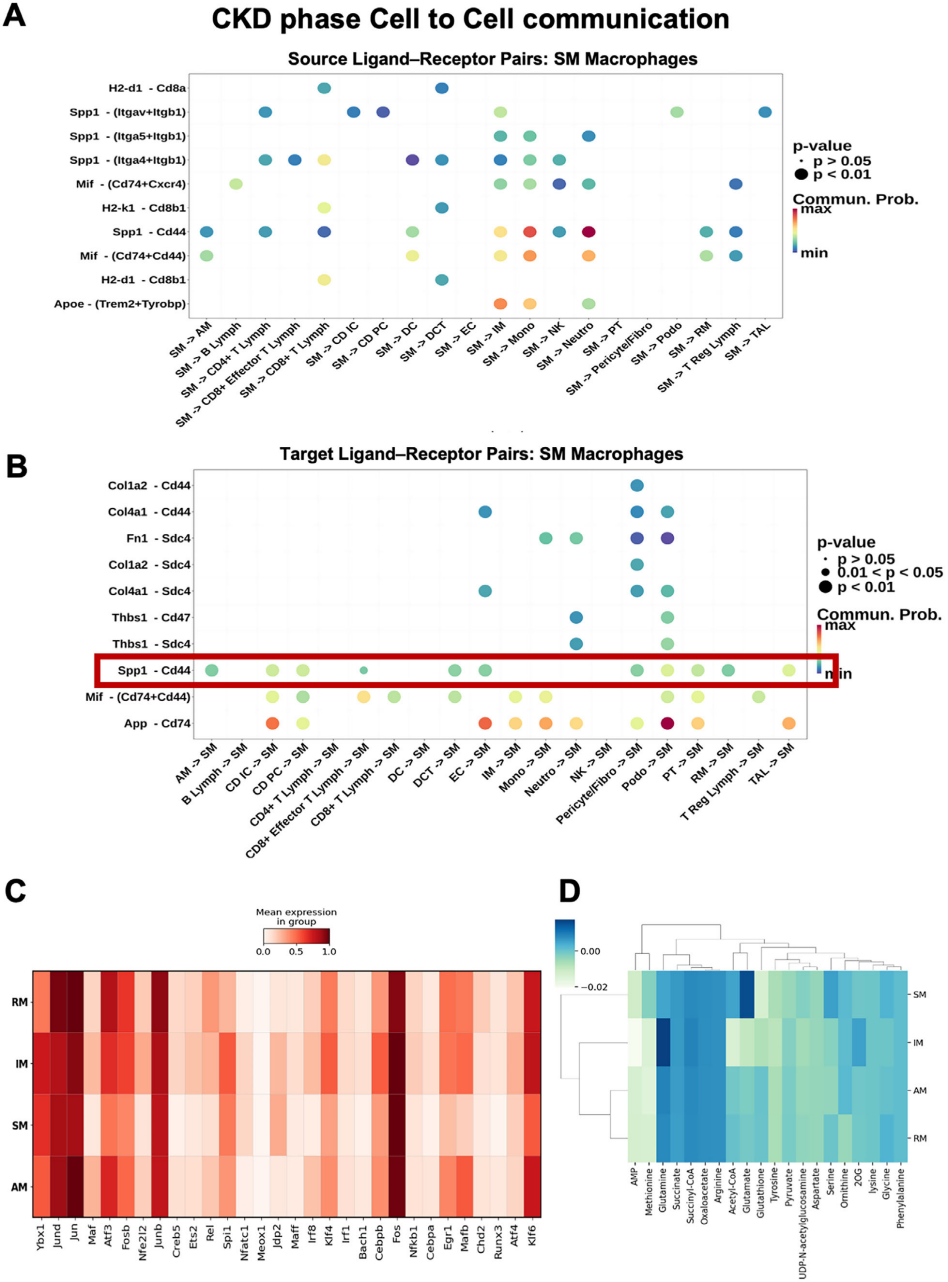
Scar‐associated macrophage–centered signaling networks, transcription regulators and metabolic reprogramming during the AKI to CKD Transition. (A) Ligand–receptor interactions (cell to cell communication centered on SM macrophages): Sending signaling from SM macrophages. Bubble plot of ligand–receptor pairs with SM as the sender and kidney subpopulations as receivers (*x*‐axis). (B) Receiving signaling to SM macrophages. With SM as the receiver, prominent inputs arise from extracellular‐matrix ligands binding *Cd44*/*Sdc4* on SM (e.g., *Spp1*–*Cd44*, *Col1a2*/*Col4a1*‐*Cd44*, *Fn1*‐*Sdc4*, *Col1a2*/*Col4a1*‐*Sdc4*, *Thbs1*‐*Cd47*/*Sdc4*). Bubble size reflects significance, color indicates communication probability. (C) Heatmap of transcription regulator expression across RM, IM, SM, and AM subpopulations(columns). Rows list Transcription Factors. Color denotes the mean normalized expression within each subcluster. (D) Heatmap of metabolic pathway enrichment, profiling major metabolic pathway intermediates among macrophage subsets.

To clarify the role of SM within the CKD microenvironment, we performed comprehensive ligand–receptor analysis. During the CKD phase, SM subpopulations functioned as key hubs in the renal immune network (Figure 3A). As senders, SM exhibited broad outputs to multiple recipient cell types, with the strongest and most significant interactions involving IM and NK cells. As receivers, SM are major targets of signaling originating from pericyte/fibroblast populations, endothelial cells, proximal tubular cells, and other immune or nonimmune cell types (Figure 3B).

Across both directions, the *Spp1*–*Cd44* axis emerged as one of the most dominant ligand–receptor pathways (*p* < 0.01), recurrently connecting SM with parenchymal and stromal compartments. Consistent with their transcriptional profile, SM themselves express *Spp1* and can participate in autocrine *Spp1*–*Cd44* signaling; however, the modeled communication probabilities indicate that Spp1 ligands are produced predominantly by tubular cells and stromal cells, whereas *Acp5*/TRAP5^+^ SM expressed *Cd44* and received strong incoming *Spp1* signals. Thus, SM are positioned not only as profibrotic effectors but also as major recipients of *Spp1* cues from the damaged tissue environment, with the capacity for additional autocrine reinforcement. Additional enriched pairs included *Col1a2*/*Col4a1*‐*Sdc4* and *Thbs1*‐*Sdc4*/*Cd47*, further supporting a role for SM in ECM remodeling and profibrotic signaling.

Together, these results indicate that SM engages in extensive bidirectional communication across immune and stromal compartments, with *Spp1*–*Cd44* serving as a dominant signaling axis that amplifies tissue remodeling and inflammation, thereby driving fibrotic progression in CKD.

### 2.5 Subset‐Specific Transcriptional and Metabolic Pathways Underlie Macrophage Roles in Inflammation and Fibrosis

To further delineate the transcriptional regulatory landscape of macrophage subpopulations, we also performed comparative analysis of key transcription factor expression across AM, SM, IM, and RM subclusters (Figure 3C). The heatmap demonstrates that SM exhibits lower expression of several AP‐1 family members, including *Jund*, *Jun*, and *Fosb*, as well as stress‐ and activation‐related transcription factors such as *Mafb*, *Atf3*, *Klf6*, and *Nfe2l2*. Additionally, SM showed increased expression of *Nfatc1* and *Jdp2*, both of which are implicated in tissue remodeling and fibrotic gene regulation. This distinct transcription factor signature supports the functional specialization of SM and reinforces their proposed role in driving matrix remodeling and fibrogenesis during CKD.

To predict the metabolic state of macrophages subset, we performed single‐cell Flux Estimation Analysis (scFEA). The result (Figure 3D) highlights the functional specialization across macrophage subtypes. Classically activated M1 macrophage showed elevated intracellular glutamine, consistent with their proinflammatory role [17]. In contrast, tissue regeneration/repair‐associated macrophages exhibited increased glutamate, reflecting enhanced metabolic activity [18]. In line with these prior observations, our data demonstrate glutamate enrichment in scar‐associated macrophages, whereas IM presented increased glutamine. Given that glutaminolysis (the conversion of glutamine to glutamate and subsequently to α‐ketoglutarate) acts as a critical compensatory pathway, supplying the tricarboxylic acid (TCA) cycle with substrates to fuel mitochondrial respiration and ATP generation [19], the enrichment of glutamate in SMs likely reflects a metabolic shift to meet the increased energy demands associated with tissue remodeling and fibrosis.

Taken together, these results point to subset‐specific glutamine–glutamate circuits as metabolic drivers of macrophage behavior in inflammation and tissue remodeling, highlighting potential targets for intervention.

### 2.6 ACP5/TRAP5^+^ Macrophages are Validated in Murine Models of Kidney Injury

Building on our scRNA‐seq finding that *Acp5*, the top marker of SMs, encodes TRAP5‐a molecule previously implicated in fibrosis of lung and cardiovascular tissues [20, 21], we next sought to determine whether its induction generalizes across kidney injury models. We therefore assessed *Acp5* mRNA and TRAP5 protein expression in multiple AKI to CKD and CKD models (AAI and IRI for AKI to CKD, unilateral ureteral obstruction (UUO), and folic acid (FA) injury for CKD).

**FIGURE 4:**
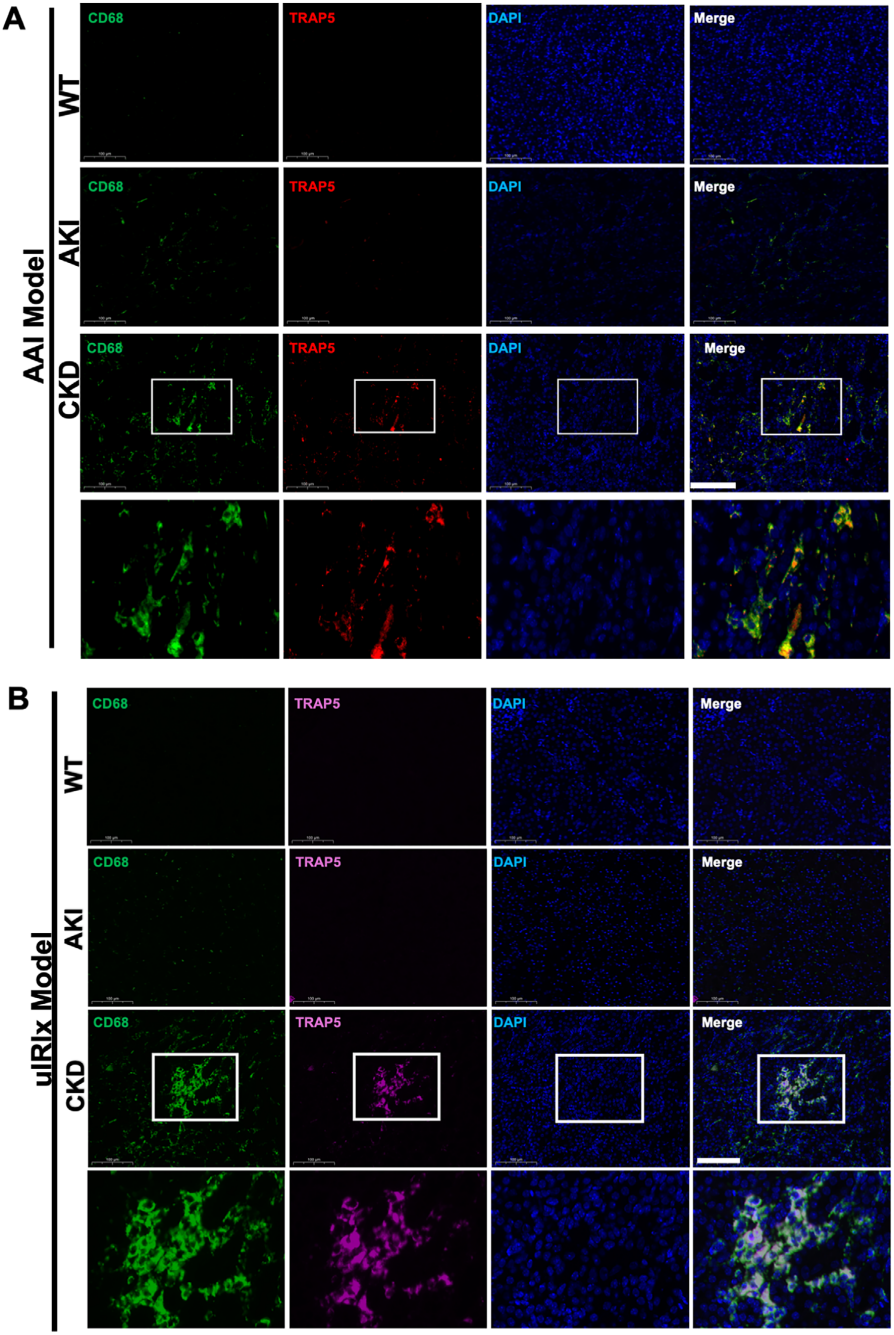
TRAP5^+^ macrophages emerge during CKD in murine AKI to CKD models. (A) Representative immunofluorescence images from the AAI model at control (CON), acute kidney injury (AKI), and chronic kidney disease (CKD) stages. Sections were stained for CD68(green), TRAP5(red), and DAPI (blue). (B) Representative immunofluorescence images from the uIRIx model at CON, AKI, and CKD. Sections were stained for CD68 (green), TRAP5 (pink), and DAPI (blue). White boxes indicate regions enlarged in the lower panels. Scale bars, 100 µm.

In the AAI‐induced AKI to CKD transition, hematoxylin and eosin (H&E) and Masson's Trichrome staining revealed increased tubular injuries and collagen deposition during the CKD phase (Figure S3A,B). Blood urea nitrogen (BUN) and serum creatinine levels peaked in the AKI phase, with partial recovery was observed in CKD phase, indicating repair and remodeling following kidney injury (Figure S3C). In line with the scRNA‐seq, *Acp5* mRNA transcript measured by quantitative polymerase chain reaction (qPCR) was significantly increased in CKD but unchanged in AKI (Figure S3D). Immunofluorescence staining confirmed abundant TRAP5^+^ CD68^+^ macrophages in CKD kidneys (Figure 4A). Furthermore, costaining of CD68 with Cathepsin K (CTSK) or MMP9 demonstrated enrichment of additional matrix‐degrading enzymes in CKD (Figure S4A).

Consistent with the AAI‐induced AKI to CKD transition model, the unilateral ischemia‐reperfusion injury with contralateral nephrectomy (uIRIx) model showed the identical trend. TRAP5^+^ macrophages were rare at day 3 but markedly increased by day 24 (Figures 4B; S5A). *Acp5* mRNA was also significant elevated at 24 days versus 3 days post uIRIx surgery (Figure S5B). In the UUO model, histological assessment revealed marked tubular injuries and substantial interstitial fibrosis, as demonstrated by H&E and Masson's trichrome staining (Figure S6A). Immunofluorescence showed a prominent accumulation of TRAP5^+^ macrophages in obstructed kidneys, with these cells displaying colocalization of TRAP5 and CD68 (Figure S6B). Similarly, in the FA‐induced renal fibrosis model, we observed pronounced tubular injuries and interstitial collagen deposition consistent with progressive fibrosis (Figure S6C). Immunostaining revealed a notable increase in TRAP5^+^ macrophages in the kidneys of FA‐treated mice, again with strong coexpression of CD68 and TRAP5 (Figure S6D).

Taken together, these data demonstrate that TRAP5^+^ macrophages are a reproducible feature of renal fibrosis in multiple murine models, implicating this macrophage subset as a potential effector of chronic inflammation and tissue remodeling in progressive kidney disease.

### 2.7 Scar‐Associated Acp5(TRAP5)^+^ Macrophages Are Also Evident in Human CKD Kidney Biopsies

**FIGURE 5:**
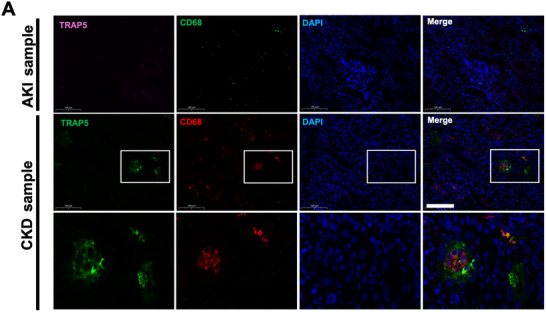
TRAP5^+^ macrophages present in human CKD but absent in AKI. (A) Representative immunofluorescence images from AKI and CKD human kidney tissue. AKI section was stained for TRAP5(green), CD68 (red), and DAPI (blue). CKD section was stained for TRAP5(red), CD68(green) and DAPI (blue). White boxes indicate regions enlarged in the lower panels. Scale bars, 100 µm.

To explore whether accumulation of Acp5(TRAP5)^+^ macrophages observed in the experimental murine models is also present in human kidney disease, we examined kidney biopsy samples from patients with AKI and CKD. Immunofluorescence staining revealed a distinct population of TRAP5^+^CD68^+^ double‐positive macrophages within areas of tubulointerstitial injury in CKD kidney samples, whereas these cells were undetectable in AKI biopsies (Figure 5A).

To corroborate these histological findings, we further analyzed scRNA‐seq data from the Kidney Precision Medicine Project (KPMP) [22]. Within the human macrophage population, expression of *ACP5* exhibited significant positive correlations with key profibrotic and remodeling markers including *COL1A1*, *MMP9*, *TGFB1*, *VIM*, and *SPP1* (Figure S7A). These association indicate that macrophage‐intrinsic *ACP5* expression closely tracks with the severity of human renal fibrosis. Furthermore, CD68^+^ macrophages coexpressing MMP9 or CTSK were observed in CKD (Figure S8A).

These findings indicate that the emergence of TRAP5^+^ scar‐associated macrophages is a conserved feature of progressive renal fibrosis in both experimental murine models and human CKD.

### 2.8 Inhibition of TRAP5 Markedly Ameliorates Renal Fibrosis Following AAI Injection

**FIGURE 6:**
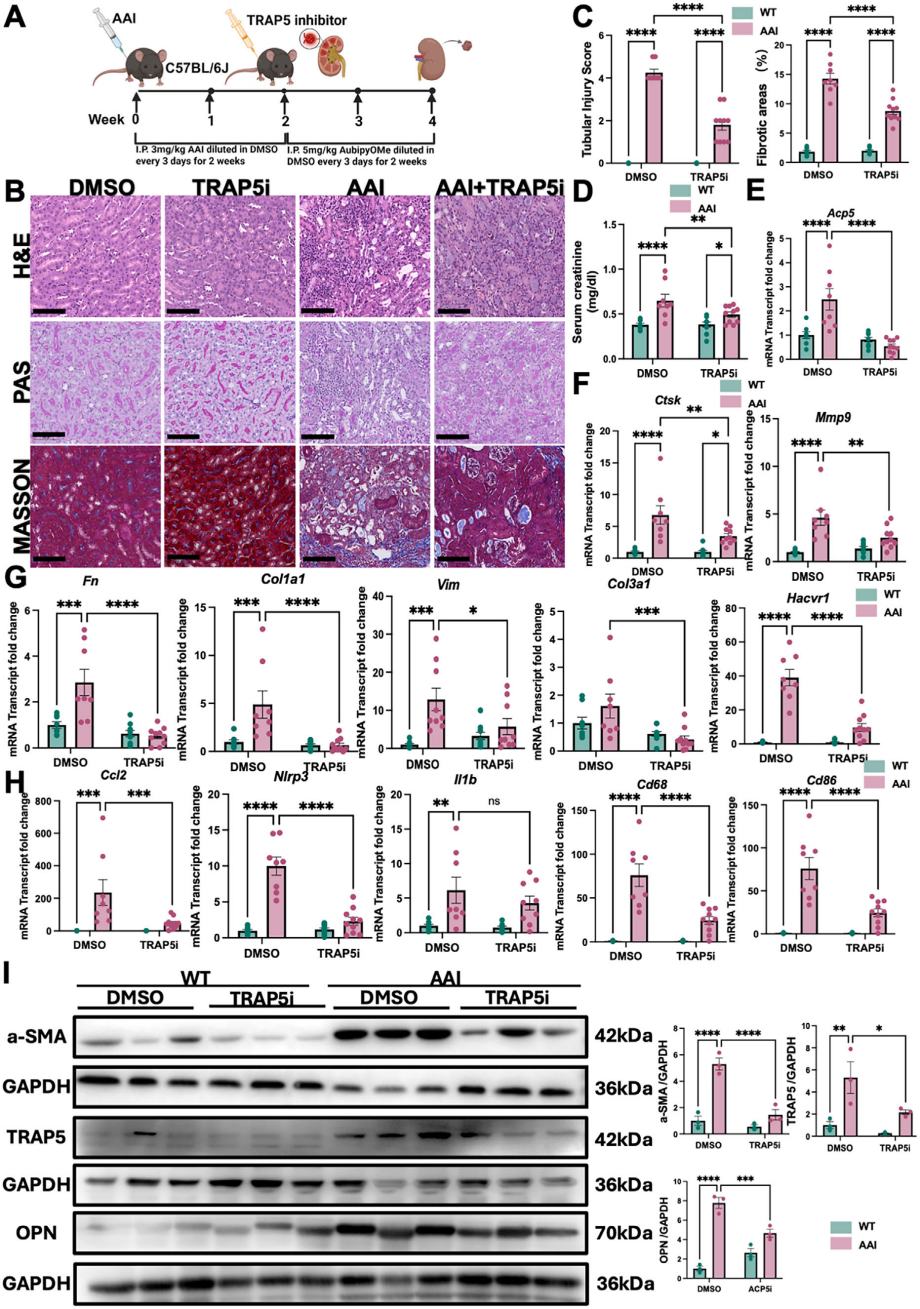
TRAP5 inhibition ameliorated AAI‐induced injury and fibrosis. (A) Schematic design of pharmacological treatment with TRAP5 inhibitor (TRAP5i) in AAI‐induced AKI to CKD transition murine model. (B) Representative images of H&E, PAS, and Masson's Trichrome staining of kidney sections from wild‐type (WT) and AAI‐injected mice with or without TRAP5i treatment. Scale bars, 100 µm. (C) Tubular injury scores and quantification of fibrotic areas. (D) Serum creatinine levels of WT and AAI‐injected mice with or without TRAP5i treatment (*n* = 8 in DMSO group, *n* = 8 in AAI+DMSO group, *n* = 10 in TRAP5i, *n* = 10 in AAI + TRAP5i). (E) Quantitative mRNA transcript of *Acp5* (*n* = 8 in DMSO group, *n* = 8 in AAI+DMSO group, *n* = 10 in TRAP5i, *n* = 10 in AAI + TRAP5i). (F) Relative mRNA transcript of matrix degrading enzyme genes (*Ctsk*, *Mmp9*) (*n* = 8 in DMSO group, *n* = 8 in AAI + DMSO group, *n* = 10 in TRAP5i, *n* = 10 in AAI + TRAP5i). (G,H) Relative mRNA transcript of fibrosis‐, injury‐, and inflammation‐related genes *(Fn*, *Col1a1*, *Vim*, *Col3a1*, *Havcr1*, *Ccl2*, *Nlrp3*, *Il1b*, *Cd68*, *Cd86*) in kidneys of wild‐type (WT) and mice subjected to AAI injections with or without TRAP5 inhibitor (TRAP5i) (*n* = 8 in DMSO group, *n* = 8 in AAI + DMSO group, *n* = 10 in TRAP5i, *n* = 10 in AAI + TRAP5i). (I) Representative Western blots of α‐SMA, TRAP5 and OPN protein in kidney tissues (*n* = 3 per group). Densitometry analysis was performed to quantify protein expression. Data are shown as mean ± SEM. Group comparisons in (C–I) were assessed by two‐way ANOVA test (*p < 0.05, **p < 0.01, ***p < 0.001, and ****p < 0.0001).

To explore the functional role of TRAP5 in kidney fibrosis, we pharmacologically inhibited TRAP5 activity in the AAI‐induced AKI to CKD model. As outlined in the experimental design (Figure 6A), mice were subjected to peritoneal injection of AAI(3 mg/kg) every 3 days for 2 weeks to induce acute tubular injuries, followed by administration of either dimethyl sulfoxide (DMSO) or TRAP5 inhibitor (TRAP5i, AubipyOMe) during the remodeling phase.

Histological analysis revealed that TRAP5 inhibition markedly attenuated renal fibrosis and tubular injury, as evidenced by H&E, periodic acid–Schiff (PAS), and Masson's trichrome staining, which showed preserved renal histology and diminished collagen deposition in the TRAP5i‐treated group (Figure 6B). As quantified, mice treated with TRAP5 inhibitor exhibited significantly reduced fibrotic area and lower tubular injury scores compared to DMSO‐treated AAI mice (Figure 6C). Consistent with histological findings, TRAP5 inhibition significantly decreased serum creatinine in mice subjected to AAI injection, indicating improved renal function (Figure 6D).

Furthermore, qRT‐PCR analysis showed robust upregulation of matrix degrading enzymes including *Acp5*, *Ctsk*, *Mmp9* mRNA in the AAI group, which were effectively suppressed by TRAP5 inhibitor treatment (Figure 6E,F). Transcriptomic profiling of fibrotic and injury markers demonstrated that TRAP5 inhibition significantly reduced the expression of extracellular matrix genes (*Fn*, *Col1a1*, *Vim*, *Col3a1*), the tubular injury marker *Havcr1*(encoding Kim‐1), and proinflammatory genes (*Ccl2*, *Nlrp3*, *Il1b*, *Cd68*, *Cd86*) (Figure 6G,H). Immunoblotting analysis further confirmed a marked reduction in α‐SMA, TRAP5, and osteopontin (OPN) (encoded by *Spp1*) levels following TRAP5 inhibition (Figure 6I).

In line with these data, CD68/OPN/TRAP5 triple immunofluorescence staining revealed abundant CD68^+^OPN^+^TRAP5^+^ macrophages in AAI+DMSO kidneys, whereas TRAP5i treatment markedly diminished OPN and TRAP5 signals (Figure S9A) and reduced the number of CD68^+^OPN^+^TRAP5^+^ cells (Figure S9B). These data indicate that TRAP5 inhibition limits the accumulation of OPN^+^TRAP5^+^ scar‐associated macrophages in vivo.

Together, these data demonstrated that pharmacological targeting of TRAP5 yields robust protection against renal fibrosis, tubular injury, and inflammation in the AAI‐induced model.

### 2.9 TRAP5 Inhibition Attenuates the Progression from AKI to CKD Following Ischemia‐Reperfusion Injury

**FIGURE 7:**
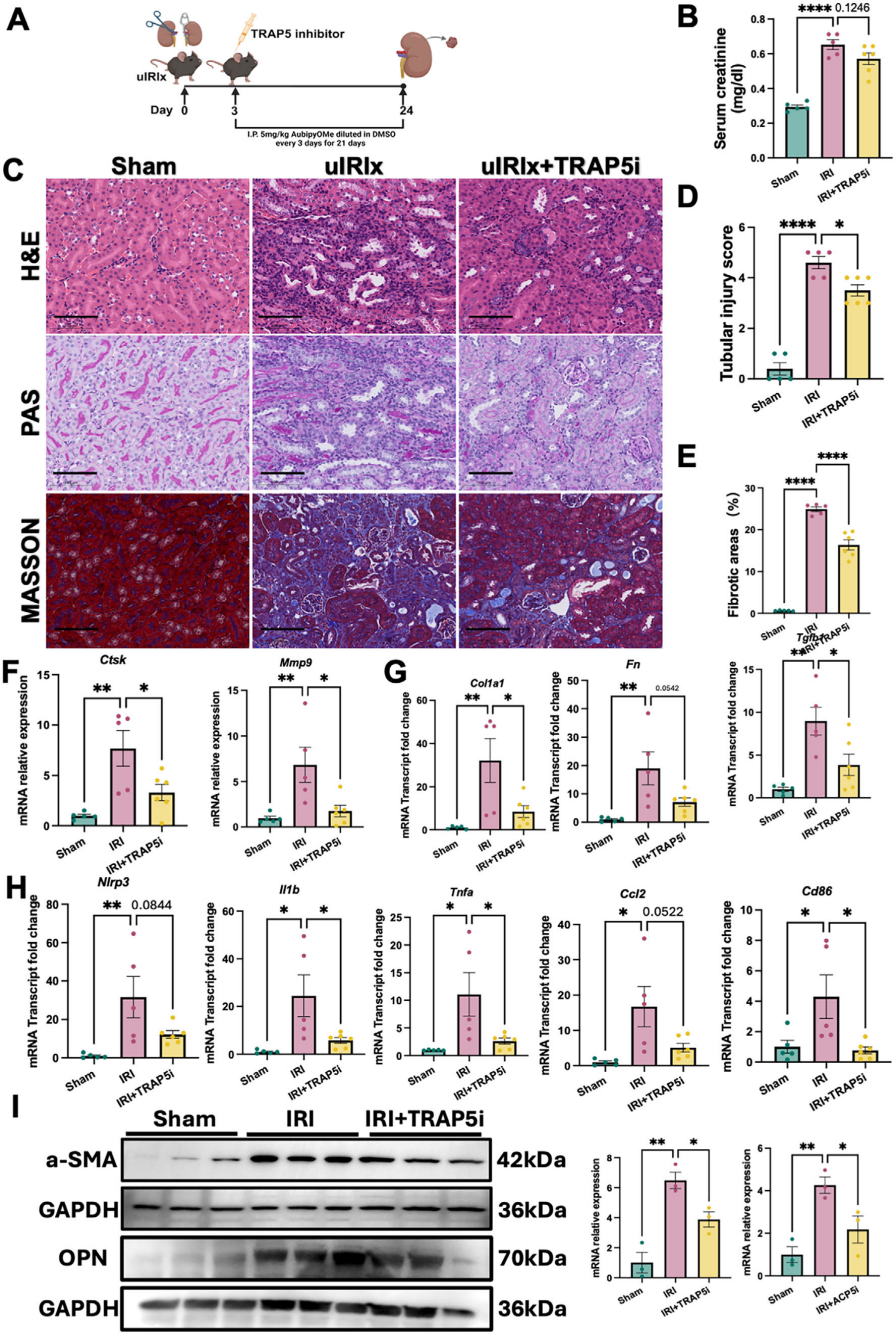
TRAP5 inhibition attenuated kidney injury and fibrosis in uIRIx‐induced AKI to CKD. (A) Schematic design of pharmacological treatment with TRAP5 inhibitor (TRAP5i) in the uIRIx‐induced AKI to CKD murine model. (B) Serum creatinine levels in sham, uIRIx and uIRIx + TRAP5i groups (*n* = 5 in WT group, *n* = 5 in uIRIx group, *n* = 6 in uIRIx + TRAP5i group). (C) Representative images of H&E, PAS, and Masson's Trichrome staining of kidney sections from sham, uIRIx, and uIRIx + TRAP5i groups. Scale bars, 100 µm. (D) Tubular injury scores (*n* = 5 in WT group, *n* = 5 in uIRIx group, *n* = 6 in uIRIx + TRAP5i group). (E) Quantification of fibrotic areas (*n* = 5 in WT group, *n* = 5 in uIRIx group, *n* = 6 in uIRIx + TRAP5i group). (F) Relative mRNA transcript of *Ctsk* and *Mmp9* (*n* = 5 in WT group, *n* = 5 in uIRIx group, *n* = 6 in uIRIx + TRAP5i group). (G) Relative mRNA transcript of fibrosis‐related genes (*Col1a1*, *Fn*, and *Tgfb1*) (*n* = 5 in WT group, *n* = 5 in uIRIx group, *n* = 6 in uIRIx + TRAP5i group). (H) Relative mRNA transcript of inflammation‐related genes (*Nlrp3*, *Il1b*, *Tnfa*, *Ccl2*, *Cd86)* (*n* = 5 in WT group, *n* = 5 in uIRIx group, *n* = 6 in uIRIx + TRAP5i group). (I) Representative Western blots of α‐SMA and OPN protein in kidney tissues (*n* = 3 per group). Densitometry analysis was performed to quantify protein expression, normalized to GAPDH. Data are shown as mean ± SEM. Group comparisons in (C–I) were assessed by one‐way ANOVA test (*p < 0.05, ** *p* < 0.01, ***p < 0.001, ****p < 0.0001).

To further elucidate the role of TRAP5 in mediating the transition from AKI to CKD, we employed a murine model of unilateral ischemia‐reperfusion injury with contralateral nephrectomy(uIRIx). TRAP5 inhibition was initiated three days postsurgery to target the maladaptive phase and renal outcomes were assessed 24 days after surgery (Figure 7A).

Consistent with the pathophysiology of uIRIx, modeling mice exhibited a significant elevation in serum creatinine, persistent tubular injuries, and pronounced interstitial fibrosis 24 days post IRI (Figure 7B–E). In contrast, administration of the TRAP5 inhibitor led to a reduction in serum creatinine, indicating preserved renal function (Figure 7B). Morphological evaluation revealed markedly reduced tubular damage and a notable decrease in interstitial collagen accumulation in TRAP5 inhibitor‐treated group compared to modeling group (Figure 7C–E). At the transcript level, qPCR demonstrated that TRAP5 inhibition effectively blunted the maladaptive upregulation of key profibrotic and proinflammatory genes characteristic of chronic post‐IRI remodeling. Specifically, transcript levels of matrix degrading enzymes(*Ctsk*, *Mmp9*) (Figure 7F), fibrosis‐related genes (*Fn*, *Col1a1*, *Tgfb1*) (Figure 7G), and inflammatory cytokines and markers (*Nlrp3*, *Il1b*, *Ccl2*, *Cd86*) (Figure 7H) were all suppressed following TRAP5 inhibition. This broad suppression of pathogenic gene expression was paralleled by a substantial reduction in α‐SMA and OPN protein abundance, as shown by immunoblot analysis (Figure 7I).

Together, these findings highlight a central role for TRAP5^+^ macrophages in driving postischemic fibrogenesis and position TRAP5 as a promising therapeutic target for halting AKI to CKD progression following ischemic kidney injury.

### 2.10 Pretreatment of TRAP5 Inhibition Also Attenuates UUO Induced Renal Fibrosis

**FIGURE 8:**
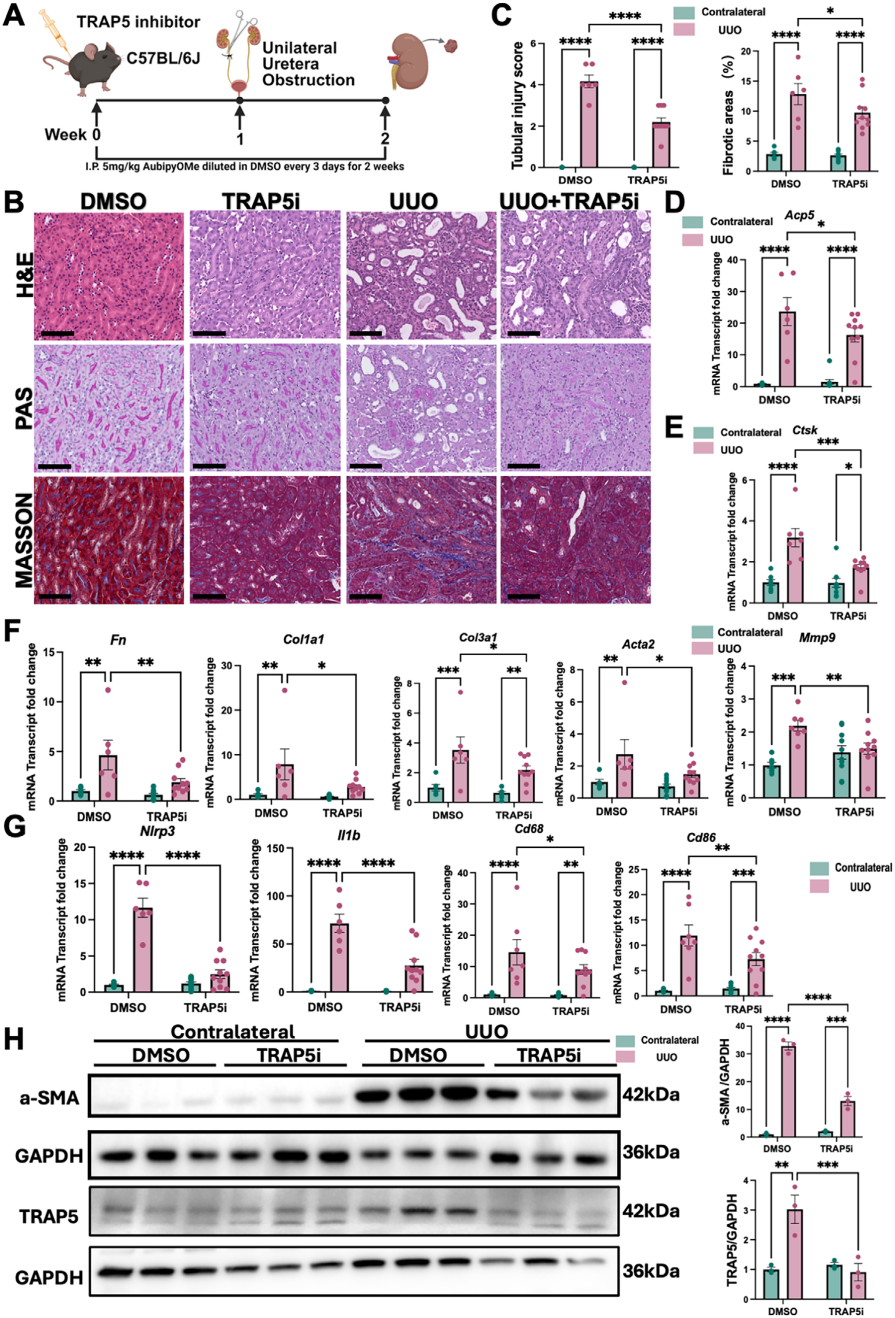
TRAP5 Inhibition pretreatment mitigated UUO induced renal fibrosis. (A) Schematic design of pharmacological pretreatment with TRAP5 inhibitor (TRAP5i) in UUO murine model. (B) Representative images of H&E, PAS, and Masson's Trichrome staining of kidney sections from contralateral unobstructed and obstructed kidneys with or without TRAP5i. Scale bars, 100 µm. (C) Tubular injury score and quantification of fibrotic areas (*n* = 6 in DMSO group, *n* = 10 in TRAP5i‐treated group). (D) Relative mRNA transcript of *Acp5*(*n* = 6 in DMSO group, *n* = 10 in TRAP5i‐treated group). (E) Relative mRNA transcript of ECM‐degrading enzymes (*Ctsk*, *Mmp9*) (*n* = 6 in DMSO group, *n* = 10 in TRAP5i‐treated group). (F) Relative mRNA transcript of fibrosis‐associated genes (*Fn*, *Col1a1*, *Col3a1*, *Acta2*) (*n* = 6 in DMSO group, *n* = 10 in TRAP5i‐treated group). (G) Relative mRNA transcript of inflammation‐associated genes (*Nlrp3*, *Il1b, Cd68, Cd86*) (*n* = 6 in DMSO group, *n* = 10 in TRAP5i‐treated group). (H) Representative Western blots of α‐SMA and TRAP5 in kidney tissues (*n* = 3 per group). Densitometry analysis was performed to quantify protein expression, normalized to GAPDH. Data are shown as mean ± SEM. Group comparisons in (C–H) were assessed by two‐way ANOVA test (*p < 0.05, **p < 0.01, ***p < 0.001, ****p < 0.0001).

To further evaluate the effect of TRAP5 inhibition in an independent murine model of kidney fibrosis, we also utilized the UUO model. As depicted in the experimental scheme (Figure 8A), mice were pretreated with or without the TRAP5 inhibitor one week prior to the UUO surgery.

Pretreatment with the TRAP5 inhibitor led to a marked attenuation of renal injury and fibrosis in the UUO model. Histological analyses using H&E, PAS, and Masson's trichrome staining presented mild tubular injuries and decreased interstitial collagen deposition in the TRAP5i‐treated group compared to UUO alone (Figure 8B). Quantitative analysis corroborated with these findings, revealing significant reductions in both tubular injury scores and fibrotic area in the TRAP5i intervention group (Figure 8C). Consistent with these histological improvements, *Acp5*, *Ctsk*, *Mmp9* mRNA expression was significantly elevated in the obstructed kidneys, but was markedly suppressed by TRAP5 inhibitor (Figure 8D,E). Inhibition of TRAP5 also resulted in downregulation of multiple fibrotic and inflammatory genes, including *Fn*, *Col1a1*, *Col3a1*, *Acta2*, *Nlrp3*, *Il1b*, *Cd68*, and *Cd86* (Figure 8F,G). Moreover, these changes were accompanied by a substantial reduction in α‐SMA and TRAP5 protein expression, as confirmed by Western blot analysis (Figure 8H).

Together, analyses of AAI, uIRIx, and UUO models demonstrated that TRAP5 inhibition consistently ameliorates renal injury, fibrosis, and inflammation, highlighting TRAP5 as a potential therapeutic target across diverse forms of progressive kidney disease.

### 2.11 TRAP5 Inhibitor Abrogates OPN‐Induced Macrophage Activation and Fibrotic Signaling Pathways In Vitro

To elucidate the cellular mechanisms underlying TRAP5 inhibition, we investigated its impact on macrophage activation, metabolism, and gene expression. Treatment of bone marrow‐ derived macrophage (BMDMs) with TRAP5 inhibitor significantly downregulated *Acp5*(TRAP5) at both mRNA and protein levels (Figure S10A,B). Additionally, TRAP5 inhibition broadly suppressed genes associated with macrophage activation and polarization, including *Spp1*, *Il10*, *Tgfb1*, *Ym1*, *Mrc1*, *Retnla*, *Mr*, *Cd68*, *Cd86*, and *Ccl2* (Figure S10A) and decreased cell viability (Figure S10C).

**FIGURE 9:**
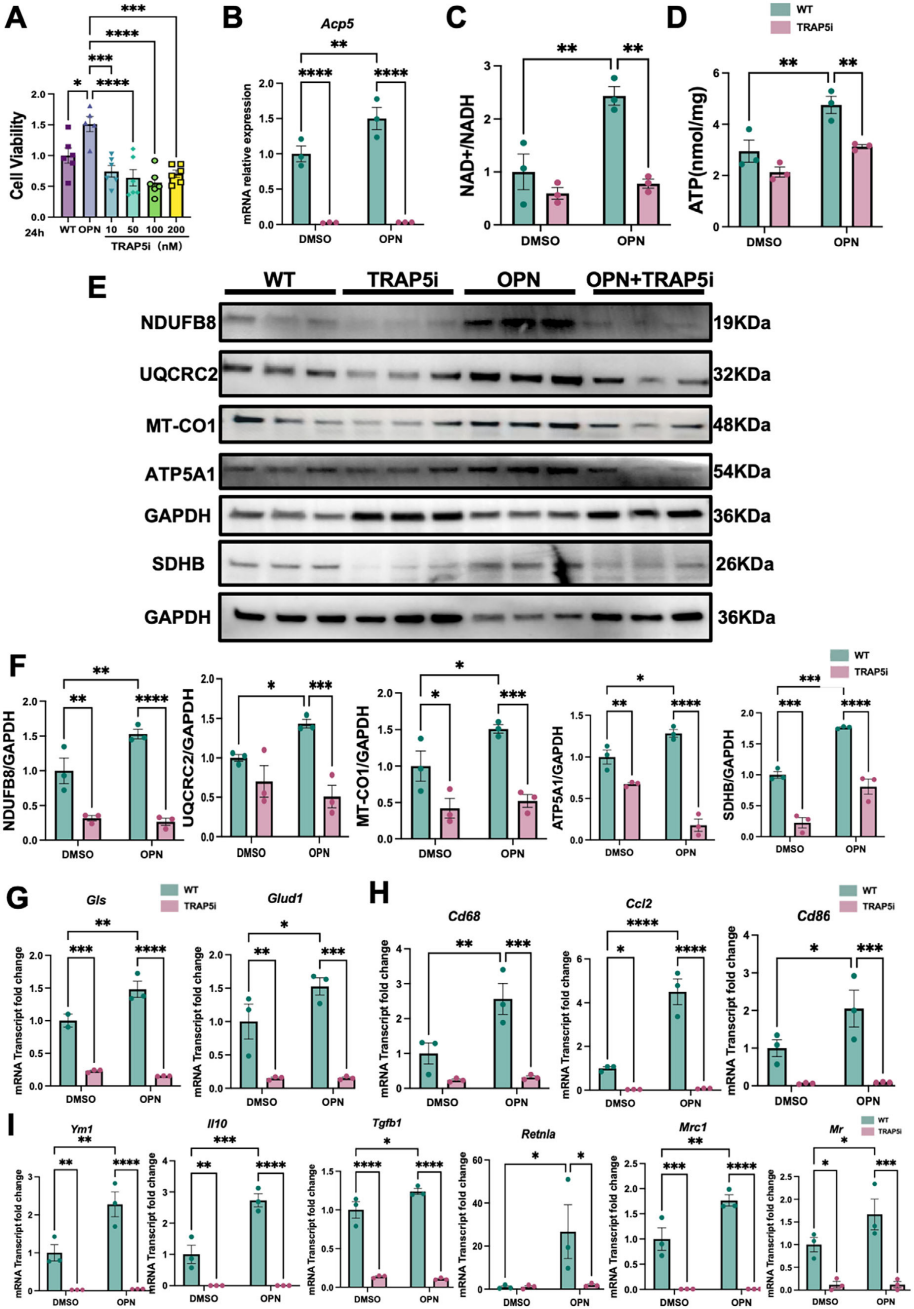
TRAP5 regulated macrophage metabolic functions and polarization in response to OPN. (A) Cell viability of BMDMs after 24 h exposure to OPN (Osteopontin) with or without TRAP5i in a dose dependent manner (*n* = 6 per group). (B) *Acp5* mRNA levels in BMDMs stimulated with OPN ± TRAP5i; (C) NAD^+^/NADH ratios in BMDMs after OPN stimulation with or without TRAP5i (*n* = 3 independent experiments per group). (D) Measurement of ATP in BMDMs treated with OPN ± TRAP5i (*n* = 3 independent experiments per group). (E) Representative immunoblots of mitochondrial respiratory chain/ATP synthase subunits (NDUFB8, UQCRC2, MT‐CO1, ATP5A1, SDHB) in BMDMs treated with OPN ± TRAP5i, GAPDH shown as a loading control. (F) Densitometry analysis was performed to quantify protein expression, normalized to GAPDH (*n* = 3 independent experiments per group). (G) Relative mRNA Transcript of glutaminolysis enzymes (*Gls*, *Glud1*) (*n* = 3 independent experiments per group). (H) Relative mRNA Transcript of *Cd68*, *Ccl2*, *Cd86* (*n* = 3 independent experiments per group). (I) Relative mRNA Transcript of *Ym1*, *Il10*, *Tgfb1*, *Retnla*, *Mrc1*, *Mr* upon OPN stimulation, following TRAP5 inhibitor treatment (*n* = 3 independent experiments per group). Data are shown as mean ± SEM. Group comparisons in (A) were assessed by one‐way ANOVA, and those in (B–D, F–I) were assessed by two‐way ANOVA. (*p < 0.05, ** *p* < 0.01, ***p < 0.001, ****p < 0.0001).

Given the results of our cell to cell communication analyses and prior evidence showing that injured proximal tubules as well as pericyte and fibroblast expresses *Spp1*(encoding osteopontin, OPN) [23, 24], we modeled this microenvironment‐derived *Spp1* signaling in vitro by stimulating BMDMs with exogenous OPN. OPN significantly increased cell viability (Figure 9A), induced *Acp5* transcripts (Figure 9B), elevated NAD^+^/NADH ratios (Figure 9C) and ATP production(Figure 9D). Pharmacological TRAP5 inhibition(TRAP5i) reduced cell viability in a dose‐dependent manner (Figure 9A), suppressed TRAP5 expression and abrogated these OPN‐induced metabolic reprogramming(Figure 9C,D).

We further investigated the effects of OPN stimulation and TRAP5 inhibition on mitochondrial respiratory chain complexes by examining the expression of key mitochondrial enzymes. In line with GO enrichment analysis, Western blot analysis revealed that OPN stimulation promoted mitochondrial oxidative phosphorylation, as indicated by increased expression of respiratory chain complexes NDUFB8 (Complex I), SDHB (Complex II), UQCRC2 (Complex III), MT‐CO1 (Complex IV), and ATP5A1 (Complex V) (Figure 9E,F). Notably, TRAP5 inhibition reduced the abundance of these subunits at baseline and blunted OPN‐induced upregulation, indicating that TRAP5 activity supports OPN‐driven increases mitochondrial oxidative phosphorylation components (Figure 9E,F).

Importantly, TRAP5 inhibition substantially attenuated the OPN‐induced expression of key metabolic enzyme genes such as *Gls* (glutaminase) and *Glud1* (glutamate dehydrogenase), which convert glutamine to glutamate and then to α‐ketoglutarate to fuel the TCA cycles (Figure 9G). OPN also increased transcripts for inflammatory cytokines (*Ccl2*, *Cd68*, *Cd86*) (Figure 9H), profibrotic mediators (*Tgfb1*), and alternative activation markers (*Ym1*, *Il10*, *Retnla*, *Mrc1*, and *Mr*); cotreatment with TRAP5 inhibitor largely normalized these responses (Figure 9I).

These findings collectively demonstrate that OPN exposure induces an *Acp5* ^+^, metabolically activated, profibrotic phenotype that closely resembles the transcriptional signature of SM macrophages, while TRAP5 inhibition abrogated these responses, highlighting TRAP5 as a potential therapeutic target for mitigating fibrosis‐associated macrophage activation.

### 2.12 TRAP5 Inhibition Suppresses WNT1 and IL4 Induced Macrophage Polarization

Finally, given the known role of IL4 as a typical M2 reparative macrophage activator and the recognized involvement of WNT signaling in inducing a profibrotic phenotype in macrophage [25, 26], we also investigated the macrophage's response to IL4 and WNT1 stimulation.

We observed that *Acp5* mRNA expression was significantly induced by combined IL4 and WNT1 stimulation, but not by IL4 alone. This response is greatly suppressed by TRAP5 inhibition (Figure S10D). Furthermore, costimulation with IL‐4 and WNT1 robustly enhanced the expression of alternative activation markers (*Tgfb1*, *Ym1*, *Mrc1*) and matrix degrading enzymes (*Mmp9*), whereas TRAP5 inhibition abrogated these responses (Figure S10E).

Collectively, these in vitro findings demonstrate that TRAP5 is essential for maintaining profibrotic and alternative activation gene expression in macrophages, and its inhibition broadly suppresses pathogenic gene expression and metabolic reprogramming.

## 3 Discussion

Our study provides novel insights into the immunological landscape of the AKI to CKD transition and identifies a previously unrecognized *Acp5* ^+^ scar‐associated macrophages subset as a central driver of renal fibrosis. Using integrated scRNA‐seq, trajectory analysis, ligand–receptor modeling, and functional studies, we show that TRAP5^+^ macrophages emerge during the remodeling/CKD phase, undergo metabolic reprogramming, and orchestrate matrix remodeling and inflammation. Importantly, pharmacological TRAP5 inhibition in multiple murine models and in vitro systems attenuates fibrosis, inflammation, and mitochondrial reprogramming, thereby preserving renal structure and function.

IRI is a classic model for studying AKI to CKD transition. During reperfusion, renal tubular epithelial cells and the microvasculature experience oxidative stress, inflammation, and endothelial dysfunction [27], ultimately leading to maladaptive repair and fibrosis. Recent scRNA‐seq studies have mapped key epithelial‐macrophage interactions in this context, including macrophage CD38‐driven NAD^+^ depletion promotes tubular senescence and injury [12], and sustained metabolic reprogramming of injured tubules that drives fibrosis via a *Cd44*‐*Fgfr2* axis [28]. Additional work has described ECM‐remodeling macrophages communicating with fibroblasts through *Igf1*‐*Igf1r* and *Fn1*‐*Cd44* signaling [9], and polyploid proximal tubule cells adopting a profibrotic transcriptional state via *Spp1* signaling [23].

In contrast, AAI‐induced AKI to CKD transition models direct nephrotoxic injury that predominantly targets proximal tubular epithelial cells, leading to DNA adduct formation and tubular cell apoptosis/necrosis [21, 29, 30], followed by interstitial inflammation and fibrosis [13, 31]. This model is particularly relevant for tubulointerstitial nephritis and repeated tubular injuries with defective repair. In this model, we identify a macrophage subset coexpressing *Acp5, Mmp9*, *Ctsk*, and *Spp1*, consistent with an ECM‐remodeling phenotype. Concordantly, prior IRI studies have reported a similar macrophage subpopulation with elevated ECM components (*Fn1*, *Spp1*), metalloproteinases (*Mmp9*, *Mmp12*, *Mmp19*), and cathepsins (*Ctsb*, *Ctsd*, *Ctsz*, *Ctsl*, and *Ctss*) in IRI‐induced AKI to CKD model [9]. Critically, our single‐cell transcriptomic and protein‐level analyses reveal that *Acp5*/TRAP5 is highly and specifically expressed in these scar‐associated macrophages, while its expression remains negligible in other renal cell types, including tubular cells and fibroblasts. These convergent findings across distinct injury paradigms support a conserved role for *Acp5* ^+^ ECM‐remodeling macrophages in chronic kidney injury.

SPP1(OPN) is an established profibrotic ligand in kidney disease [24, 32]. It is rapidly induced after kidney injury [23], promotes macrophage recruitment [33] and interstitial fibrosis [34], and correlates with CKD severity [35]. Previous kidney studies have mainly emphasized tubule to fibroblast signaling via the *Spp1*–*Cd44* axis [36]; our computational ligand–receptor analysis extends this view by predicting that *Spp1* ligands are produced predominantly by tubular and stromal cells, while *Acp5* ^+^ scar‐associated macrophages express *Cd44* and receive strong incoming *Spp1* signals. Thus, TRAP5^+^ scar‐associated macrophages are positioned not only as profibrotic effectors but also as key targets of microenvironmental SPP1 signaling.

Consistent with this directional model, OPN protein is markedly upregulated in AAI‐ and IRI‐ induced AKI to CKD kidneys and significantly reduced by TRAP5 inhibition, in parallel with reduced fibrosis. CD68/OPN/TRAP5 costaining further demonstrates that TRAP5 blockade diminishes OPN^+^TRAP5^+^ macrophages in vivo. Pharmacological TRAP5 inhibition therefore not only attenuates macrophage activation but also reshapes the OPN‐rich fibrotic microenvironment. Thus, our work extends prior macrophage‐focused fibrosis studies by identifying a TRAP5^+^OPN‐responsive scar‐associated macrophage subset and defining its metabolic reprogramming as a key driver of AKI to CKD progression.

Our OPN‐stimulation experiments in BMDMs provide a simplified in vitro model of this axis. In BMDMs, OPN induces Acp5/TRAP5 expression, enhances glutaminolysis and mitochondrial oxidative phosphorylation, and promotes profibrotic activation, whereas TRAP5 inhibition reverses these metabolic and transcriptional changes, indicating that TRAP5 activity is necessary to maintain an OPN/SPP1‐high profibrotic state. Together with the cell to cell communication analysis, these data support a framework in which OPN‐rich injured tubules and stroma fuel the pathogenic activity of TRAP5^+^ SM via the *Spp1*–*Cd44* axis during the AKI to CKD transition.

Mechanistically, although we did not directly assess TRAP5 substrates or OPN dephosphorylation in this study, our findings are compatible with previous work demonstrating that TRAP5 is an acid phosphatase that dephosphorylates OPN and that dephosphorylated OPN exhibits enhanced adhesion and migration‐promoting activity on integrin‐ or CD44‐dependent matrices, thereby modulating OPN‐dependent immune cell recruitment [37, 38, 39]. In a murine pulmonary infection model [40], loss of *Acp5* resulted in hyperphosphorylated OPN and impaired myeloid recruitment, whereas reconstitution with enzymatically active TRAP5 restored leukocyte recruitment, providing in vivo evidence that TRAP5 controls OPN phosphorylation and OPN‐ driven myeloid trafficking. Moreover, in lung fibrosis, fibroblast‐derived TRAP5 has been shown to dephosphorylate β‐catenin at Ser33/Thr41, stabilizing β‐catenin and amplifying TGF‐β‐driven profibrotic signaling [41]. Together with phosphoproteomic analyses in TRAP5‐overexpressing cancer cells linking TRAP5 to adhesion/ECM signaling, CD44/TGFβ pathways, and mitochondrial processes [42], these observations support a working model in which OPN upregulates TRAP5 in scar‐associated macrophages and in turn, TRAP5 dephosphorylates OPN and possibly other ECM or signaling proteins, thereby amplifying OPN/CD44 signaling and coupling it to mitochondrial metabolic reprogramming. In this positive feed‐forward loop, OPN and TRAP5 reinforce each other to sustain a high ‐OXPHOS, profibrotic state in scar‐associated macrophages.

Studies outside the kidney further support an OPN‐mitochondria axis. OPN has been shown to maintain macrophage proteostasis and mitochondrial function, as OPN depletion in BMDMs impairs proteostasis and triggers mitochondria‐mediated apoptosis [43]. OPN can induce mitochondrial biogenesis in de‐adherent cancer cells [44], and reprogram macrophages in aging adipose tissue into a senescence‐like, efferocytosis‐defective state that is reversible with OPN blockade [45]. These extra‐renal observations align with our data and suggest that OPN‐driven mitochondrial activation in macrophages is a broader phenomenon, with TRAP5 as an important downstream effector.

TRAP5 itself is predominantly expressed in macrophages and has been implicated in tissue remodeling across multiple organs. Fibroblast‐derived TRAP5 promotes pulmonary fibrosis through β‐catenin signaling [20, 41] and facilitates immune cell recruitment [40, 46], while macrophage TRAP5 supports alveolar macrophage proliferation via β‐catenin pathways [47]. In cardiac studies, TRAP5 has been associated with remodeling and fibrosis [21, 48]. Our work extends these observations to the kidney, demonstrating an expansion of TRAP5^+^ macrophages in multiple injury models and showing that TRAP5 blockade consistently ameliorates fibrosis, inflammation, and renal dysfunction. Together, these data establish TRAP5 as a disease‐promoting effector of scar‐associated macrophages and a compelling therapeutic target.

We also characterized these scar‐associated macrophages by their coexpression of CTSK and MMP9, two key mediators of ECM remodeling. CTSK is a cysteine protease that degrades fibrillar collagen [49], and has been implicated in renal and pulmonary fibrosis [50, 51]. Similarly, MMP9 is upregulated in CKD‐associated macrophages and contributes to aberrant ECM deposition [31, 52, 53, 54]. Our data indicate that MMP9‐expressing macrophages are predominantly associated with chronic tissue remodeling and fibrotic stages rather than acute inflammatory responses, further supporting a model in which TRAP5^+^/CTSK^+^/MMP9^+^ macrophages act as specialized ECM‐remodeling cells during CKD progression.

Methodologically, the use of single‐cell variational inference (scVI) for data harmonization is another strength of this study. Compared with alternative integration methods like Harmony, scVI integrates datasets across experimental conditions by explicitly modeling single‐cell gene expression distributions, thereby better preserving critical biological signals and improving the interpretation of cellular dynamics. This approach yields more reliable downstream differential and trajectory analyses, which is particularly important when dissecting subtle state transitions in macrophage subsets across distinct injury models [55].

Despite the robust findings across multiple injury models, this study has several limitations. First, while our scRNA‐seq and immunostaining analyses demonstrate that Acp5/TRAP5 is predominantly and highly expressed within scar‐associated macrophages, pharmacological inhibition cannot entirely exclude potential systemic effects on other minor cell populations. Consequently, we acknowledge the critical necessity for future studies utilizing macrophage‐specific *Acp5* conditional knockout mice or macrophage‐targeted AAV‐mediated TRAP5 knockout or knockdown models to definitively validate the macrophage‐intrinsic requirement of TRAP5 in renal fibrosis. Second, the precise upstream receptors and signaling cascades by which OPN activates TRAP5, and the direct molecular substrates that link TRAP5 to metabolic reprogramming, remain to be fully elucidated. Receptor‐level perturbations and unbiased phosphoproteomic/substrate‐trapping strategies will be needed to fully dissect the OPN–TRAP5 axis. Third, while our data suggest a functional link, we have not yet performed *Spp1* loss‐of‐function studies in our AKI to CKD models. Future experiments combining tubular or stromal *Spp1* deficiency with TRAP5 inhibition will be important to validate this axis in vivo. Finally, although we confirmed the expansion of TRAP5^+^ macrophages in human CKD biopsies and identified positive correlations with fibrotic markers in the KPMP dataset, larger prospective clinical cohorts are required to establish the definitive prognostic and therapeutic relevance of this macrophage subset in clinical practice.

In conclusion, we identify a previously unrecognized *Acp5^+^* scar‐associated macrophage population that emerges during the AKI to CKD transition, undergoes metabolic reprogramming and orchestrate fibrotic and inflammatory responses. Our functional studies demonstrate that pharmacological inhibition of TRAP5 robustly mitigates renal fibrosis and maladaptive remodeling across multiple disease models. Together, these findings establish TRAP5 as a central mediator of scar‐associated macrophage pathology and highlight the OPN‐TRAP5 axis as a promising therapeutic target to halt or slow the progression from AKI to CKD.

## 4 Materials and Methods

### 4.1 Experimental Animals and CKD Model Induction

Male C57BL/6 mice, aged 8 to 10 weeks, were used to establish four distinct models for the purpose of investigating the roles of macrophages. All animal procedures were approved by the Institutional Animal Care and Use Committee (IACUC) of Shanghai University of Traditional Chinese Medicine and conducted following institutional guidelines (YYLAC18905, YYLAC‐2019‐052‐01, and PZSHUTCM2506260014).

(1) Aristolochic Acid I‐induced AKI to CKD transition model: AKI was induced by intraperitoneal injections of AAI (Sigma, USA) at a dose of 3 mg/kg, administered every 3days over 2 weeks, as previously described [14], followed by a two‐week cessation to allow for CKD development. Control mice received equivalent volumes of vehicle solution on the same schedule. Experimental groups consisted of control (*n* = 6), AKI phase (*n* = 6), and CKD phase (*n* = 6) mice.

(2) Ischemia‐Reperfusion Injury with contralateral nephrectomy (uIRIx) Model: The uIRIx model was performed as previously described [56]. Briefly, the right kidney was surgically removed (uninephrectomy) via a right flank incision. Subsequently, the left kidney was externalized through a left flank incision, and the left renal artery and vein were clamped using an FST microvascular clamp (18055‐04) for 27 min. During ischemia, the kidney was returned to the abdominal cavity, and the incision was covered with saline‐soaked gauze. In the sham group, the left kidney was exposed but not clamped. Kidneys were harvested 24 days after surgery, with a sample size of *n* = 6 per group.

(3) Folic Acid Nephropathy Model: CKD was induced using a folic acid nephropathy model, in which mice received a single intraperitoneal injection of folic acid (250 mg/kg, dissolved in 300 mm NaHCO3) [57]. Animals were sacrificed seven days postinjection. Each group consisted of six mice (control, *n* = 6; CKD, *n* = 6).

(4) UUO Model: The UUO model was established as previously described [58]. Briefly, a small incision was made through the left flank muscle to expose the left kidney, and the left ureter was ligated near the renal hilum with a 4‐0 silk suture to achieve complete obstruction. The right kidney was exposed but not ligated, serving as an internal control. Both the obstructed and contralateral kidneys were removed seven days after surgery (*n* = 6 per group).

(5) Perioperative Care: For all surgical procedures, mice were anesthetized with 2% pentobarbital (40 mg/kg) and placed on a heating pad. Intraoperative fluid replacement was provided by subcutaneous injection of 40 mL/kg 0.9% saline. For postoperative analgesia, buprenorphine was administered subcutaneously.

(6) Pharmacological Intervention with TRAP5 Inhibitor: To evaluate the functional role of TRAP5, the selective TRAP5 inhibitor AubipyOMe (5 mg/kg, Sigma) was administered intraperitoneally in three models as follows:

I. AAI Model: AAI model. AKI was induced with intraperitoneal injections of AAI (3 mg/kg) every 3 days for 2 weeks. During the subsequent CKD remodeling phase, the TRAP5 inhibitor AubipyOMe was administered intraperitoneally on days 1, 3, 5, 7, 9, and 11 of the remodeling phase (corresponding to experimental days 15, 17, 19, 21, 23, and 25). Mice were euthanized at week 4 (day 28). Group sizes: control (*n* = 8), AAI + vehicle (*n* = 8), TRAP5i only (*n* = 10), and AAI+TRAP5i (*n* = 10).

II. uIRIx Model: Mice underwent unilateral ischemia–reperfusion injury with contralateral nephrectomy (uIRIx). The TRAP5 inhibitor (AubipyOMe) was initiated on postoperative day 3 and subsequently administered every 3 days via intraperitoneal injection until sacrifice. Kidneys were collected on postoperative day 24. Groups: Sham (*n* = 5), uIRIx + vehicle (*n* = 5), and uIRIx + TRAP5i (*n* = 6).

III. UUO Model: Mice received three prophylactic injections of TRAP5 inhibitor (AubipyOMe) during the week preceding surgery, followed by additional doses on postoperative days 1, 3, and 5. Mice were sacrificed on day 7 postsurgery. Group sizes: vehicle (*n* = 6) and TRAP5i (*n* = 10). For each mouse, the contralateral unobstructed kidney served as an internal control (Con or Con + TRAP5i), while the obstructed kidney was analyzed as UUO or UUO + TRAP5i.

### 4.2 Single‐Cell RNA Sequencing

The AAI‐induced AKI and CKD models were also utilized for scRNA‐seq. Kidney tissues from control, AKI, and CKD phase mice were harvested and immediately processed to obtain single‐cell suspensions. scRNA‐seq libraries were prepared using the Chromium Single Cell 3′ Reagent Kits v2 (10× Genomics) following the manufacturer's protocol. Libraries were sequenced on an Illumina NovaSeq 6000 platform, generating 150 base pair paired‐end reads. Sample sizes were as follows: control group (*n* = 1), AKI phase (*n* = 2), CKD phase (*n* = 2). Single‐cell transcriptome sequencing were conducted by OE Biotech Co., Ltd (Shanghai, China).

### 4.3 Data Preprocessing and Analysis

Raw sequencing data were processed using Cell Ranger v7.2.0 (10× Genomics) and aligned to the GRCm39 mouse transcriptome reference using the STAR aligner; unique molecular identifiers (UMIs) were assigned, and gene expression matrices were generated for each sample; quality control steps included filtering out cells with mitochondrial gene content exceeding 30%, correcting for ambient RNA contamination using CellBender v0.3.2, and detecting doublets with scDblFinder v3.19; cells expressing fewer than 200 genes and genes expressed in fewer than three cells were excluded, resulting in a dataset comprising 39 345 single cells and 21 878 genes for downstream analysis; batch correction and data integration across samples were subsequently performed using scVI (scVI‐tools v1.3.1), training a VAE on 8000 highly variable genes with sample as the batch key to harmonize embeddings while preserving biological variation for downstream subclustering and visualization.

#### 4.3.1 Identification of Marker Genes and Cell Types

Marker genes for each cluster were identified using the rank_genes_groups function in Scanpy v1.10.1 with the *t*‐test_overestim_var method, selecting the top 300 genes per cluster; clusters were annotated based on the expression of canonical marker genes, which were referenced from the MouseWK database (https://esbl.nhlbi.nih.gov/Databases/MouseWK/WKMarkers.html). Major cell types such as Proximal tubule cells (*Lrp2*, *Slc34a1*), Distal convoluted tubule cells (*Slc12a3*), Thick ascending limb cells (*Slc12a1*), Collecting duct intercalated cells (*Atp6v1b1*, *Hmx2*), Collecting duct principal cells (*Aqp2*, *Avpr2*), Podocytes (*Wt1*, *Nphs1*), Pericyte/fibroblasts (*Acta2*, *Col1a1*), endothelial cells (*Plvap*, *Emcn*), B lymphocytes (*Cd79a*, *Cd79b*), T regulatory lymphocytes (*Foxp3*), CD8^+^ T lymphocytes (*Cd8a*, *Cd8b1*), CD8^+^ Effector T lymphocyte (*Cd3e*), CD4^+^ T lymphocytes (*Cd4*, *Tcf7*), B lymphocyte(*Cd79a*, *Cd79b*), Dendritic cells (*Cd209a*, *Clec10a*), Neutrophils (*Lyz2*, *Hp*, *F13a1*), Natural killer cells (*Gzma*, *Gzmb*), Macrophage (*C1qa*, *C1qb*), and Monocyte (*Hp*, *F10*) were identified; further analysis was conducted using the same normalization and integration approach to subcluster Macrophage, identifying specific subpopulations within these cell types.

#### 4.3.2 Heatmap Generation

Gene expression patterns across different cell clusters were visualized using the Scanpy package; starting with the processed scRNA‐seq data, marker genes were identified for each cluster using the rank_genes_groups function, and the top differentially expressed genes were selected to create heatmaps using the sc.pl.rank_genes_groups_heatmap function, effectively illustrating distinct gene expression profiles for each cell cluster.

#### 4.3.3 RNA Velocity

To investigate the dynamic transcriptional landscapes of single cells, RNA velocity analysis was performed using the scVelo package [59]. This method extends conventional RNA velocity estimation by leveraging a dynamical modeling framework, which enables the characterization of transient cellular states based on both spliced and unspliced transcript abundances. Following standard quality control and preprocessing, spliced and unspliced RNA counts were integrated with precomputed low‐dimensional embeddings to ensure accurate representation of cell states. The scVelo algorithm infers gene‐specific kinetic parameters and estimates RNA velocity vectors for individual cells, capturing the direction and magnitude of ongoing transcriptional changes. These velocity estimates were projected onto the existing embedding space, allowing for the visualization of putative developmental trajectories and lineage hierarchies across distinct cell populations.

#### 4.3.4 Gene Ontology Pathway Analysis

Gene ontology pathway analysis was performed using the gseapy package. DEGs from the four macrophage subclusters were identified and subjected to enrichment analysis via the Enrichr module. Significantly overrepresented GO terms were determined based on an adjusted false discovery rate (FDR) threshold of <0.05. Visualization of the top enriched GO pathways provided mechanistic insights into the biological processes underlying the transition from AKI to CKD, highlighting key molecular pathways associated with disease progression.

#### 4.3.5 Trajectory Analysis

To explore cellular differentiation and dynamic changes over time, trajectory analysis was performed using Slingshot2 and Monocle3; highly variable genes were selected, dimensionality reduction was achieved with principal component analysis (PCA), and a neighborhood graph and UMAP embedding was computed; pseudo time trajectories were calculated with Slingshot2 and Monocle3, ordering cells along developmental trajectories and identifying state transitions; differential expression analysis highlighted genes contributing to these transitions, with visualizations including UMAP plots of pseudo time and marker gene expression, as well as dot plots and heatmaps illustrating gene expression patterns across different trajectory branches [60, 61].

#### 4.3.6 Volcano Plot

Differential expression (DE) at the single‐cell level was performed in Scanpy using a Wilcoxon rank‐sum test. We report multiple‐testing–corrected *p*‐values (FDR). Genes were considered significant at |log_2_FC| ≥ 1 (FC ≥ 2) and FDR < 0.01. Volcano plots display −log_10_(raw *p*‐values) for visual dynamic range, while statistical significance and gene labeling are based on FDR thresholds.

#### 4.3.7 Cell to Cell Communication Analysis

Cell–cell communication was analyzed using CellChat2; the normalized gene expression matrix from scRNA‐seq data was used to create a CellChat object; preprocessing included identifying overexpressed genes and potential interactions, and communication probabilities were calculated to infer potential ligand–receptor interactions; low‐confidence interactions were filtered out, and pathway‐level communication probabilities were inferred and visualized using circle and bubble plots; the information flow for each signaling pathway was calculated and compared across control, AKI, and CKD phases to understand how cell‐to‐cell communication evolves during disease progression.

#### 4.3.8 Gene Regulatory Network Analysis

The pySCENIC workflow was employed to infer gene regulatory networks (GRNs) from scRNA‐seq data; gene coexpression modules were identified using the GRNBoost algorithm, detecting sets of genes with correlated expression across cells; transcription factors and their target genes (regulons) were predicted by scanning promoter regions of coexpressed genes for known TF‐binding motifs, linking modules to potential regulators; the activity of each regulon was quantified per cell using the AUCell method, which computes the Area Under the Curve (AUC) to measure target gene expression within individual cells; results were visualized using heatmaps to highlight transcription factor activity across different cell states.

#### 4.3.9 Single‐Cell Flux Estimation Analysis

Single‐cell Flux Estimation Analysis was utilized to estimate metabolic flux activities at the single‐cell level by integrating scRNA‐seq data with known metabolic networks; gene expression data were mapped onto predefined metabolic pathways, including glycolysis, the TCA cycle, and amino acid metabolism; metabolic fluxes were inferred by optimizing the consistency between gene expression and metabolic activity, resulting in predicted metabolite levels for individual cells; heatmaps were generated to depict the relative abundance of metabolites across different conditions, highlighting metabolic shifts between cell populations.

#### 4.3.10 KPMP Single‐Cell RNA‐Seq Correlation Analysis

To assess the relationship between macrophage *ACP5* and kidney fibrosis, we analyzed the publicly available KPMP single‐cell RNA‐seq dataset (Seurat format files downloaded from the KPMP data portal). For each kidney sample, we first identified annotated macrophage subsets and calculated the mean *ACP5* expression across macrophages. In parallel, whole‐tissue expression of extracellular matrix and profibrotic genes (*COL1A1*, *MMP9*, *TGFB1*, *VIM*, *SPP1*) was obtained by averaging log‐normalized expression across all cells from the same sample. These sample‐level values were then used to compute Spearman's rank correlation coefficients between macrophage *ACP5* expression and each fibrosis‐related gene, and the correlation coefficient (*r*) together with the corresponding *p* value were reported.

### 4.4 Histology and Immunofluorescence Staining

Kidney tissues were fixed in 4% paraformaldehyde, embedded in paraffin, and sectioned into 4 µm‐thick serial slices. After deparaffinization, sections from control, AKI, and CKD phase mice were stained with H&E, PAS, and Masson's Trichrome according to standard protocols. Tubular injury was scored semiquantitatively. Injury features included tubular dilation, tubular atrophy, tubular cast formation, vacuolization, and degeneration, and were graded as follows: Score 0, no tubular injury; Score 1, <10% of tubules injured; Score 2, 10%–25% of tubules injured; Score 3, 25%–50% of tubules injured; Score 4, 50%–74% of tubules injured; Score 5, >75% of tubules injured. Masson's Trichrome–positive fibrotic area was quantified in cortical regions using ImageJ by color‐threshold segmentation and expressed as percentage of total analyzed area.

For immunofluorescence staining, paraffin‐embedded sections from mouse kidneys and human kidney biopsies were deparaffinized, rehydrated, and subjected to antigen retrieval in citrate buffer (pH 6.0). After blocking with 5% bovine serum albumin, sections were incubated overnight at 4°C with primary antibodies against CD68 (Servicebio, GB113109), TRAP5/ACP5 (Abcam, ab191406), OPN (Proteintech, 22952‐1‐AP), CTSK (Abcam, ab187647), and MMP9 (Boster Bio, PB9669). After washing, sections were incubated with appropriate fluorescently labeled secondary antibodies, and nuclei were counterstained with DAPI. Images were acquired using a fluorescence microscope. For quantitative analyses, positive cells were counted in 10 nonoverlapping 100× high‐power fields per section; the mean number of positive cells per 100× field was calculated for each mouse and these per‐mouse means were used for statistical comparison between groups. ImageJ software was used for all morphometric quantifications. Studies were approved by the Research Ethics Review Committee of Yueyang Hospital of Integrated Traditional Chinese and Western Medicine, and written informed consent was obtained from all participants.

### 4.5 Isolation and Culture of BMDMs

As previously described [58], BMDMs were isolated from wild‐type mice. Bone marrow cells were flushed from femurs and tibiae, plated at a density of 1 × 10^6^ cells per mL, and cultured in RPMI 1640 medium (BasalMedia, China) supplemented with 20% L929 cell‐conditioned supernatant, 15% fetal bovine serum (FBS), and 1% penicillin/streptomycin. The culture medium was replaced on days 1 and 4. On day 7, differentiated BMDMs were treated for 48 h with following: OPN (1 µg mL^−1^, PeproTech), IL‐4 (20 ng mL^−1^, PeproTech), and Wnt1 (1 µg mL^−1^, PeproTech), with or without TRAP5 inhibitor‐ AubipyOMe (100 nm,Sigma).

### 4.6 Real‐Time PCR

Total RNA was extracted from renal tissues or cultured cells using an RNA extraction kit (Vazyme, China), followed by cDNA synthesis using a reverse transcription kit. Quantitative PCR was performed using the ABI 7500 instrument and SYBR Green Master Mix (Vazyme, China) with gene‐specific primers listed in Table S1. Relative mRNA expression levels were calculated using the 2^−ΔΔCt^ method, where ΔΔCt = (Ct_target − Ct_housekeeping).

### 4.7 Western Blot

Proteins were extracted from renal tissues or cells using RIPA lysis buffer supplemented with phosphatase and protease inhibitor cocktails. Protein concentrations were determined and equalized with the Epizyme BCA protein assay kit. Equal amounts of protein were separated by 4%–20% sodium dodecyl sulfate‐polyacrylamide gel electrophoresis (SDS‐PAGE) gradient gels and transferred to PVDF membranes. Membranes were incubated overnight at 4°C with primary antibodies against α‐SMA (CST, 19245T), OPN (Proteintech, 22952‐1‐AP), TRAP5 (Abcam, ab191406), NDUFB8(CST, 73951T), UQCRC2(CST, 99258T), COX1/MT‐CO1(CST, 55159T), SDHB (CST, 92649T), ATP5A1(CST, 18023S), or GAPDH (Epizyme, LF205S), followed by incubation with appropriate secondary antibodies for 1 h at room temperature. Immunoreactive bands were visualized using a chemiluminescence imaging system, and band intensities were quantified with Image J software.

### 4.8 Cell Viability Measurement

Primary BMDMs were seeded in 96‐well plates and treated for 24 h with the following: DMSO (control), or OPN (1 µg mL^−1^)), or AubipyOMe (10, 20, 50, 100, or 200 nm) plus OPN (1 µg mL^−1^). Following treatment,10 µL of CCK‐8 solution (BioAgrio, China) was added to each well and incubated for 2 h. Absorbance at 450 nm was measured with a SpectraMax i3x fluorometer (Molecular Devices), and cell viability was calculated accordingly.

### 4.9 Determination of Cellular Mitochondrial Function

BMDMs were seeded at 1 × 10^5^ cells per well in 6‐well plates and treated for 48 h under the following conditions: Control, TRAP5 inhibitor (100 nm), OPN (1 µg mL^−1^), or OPN (1 µg mL−^1^) plus TRAP5 inhibitor (100 nm). The NAD^+^/NADH ratios were determined using Enhanced NAD^+^/NADH Assay Kit with WST‐8 (Beyotime Biotechnology), following the manufacturer's protocols. Absorbance was measured at 340 nm using a microplate reader (LABSELECT). ATP concentrations were measured using the Enhanced ATP Assay Kit (Beyotime Biotechnology), with luminescence detection on a SpectraMax i3x fluorometer (Molecular Devices).

### 4.10 Statistical Analysis

Statistical analysis was performed with GraphPad Prism version 10.0. Data were presented as Mean ± SEM. The data were assessed for Gaussian distribution and homogeneity of variances before further analysis. Log transformation was utilized when the variances were not sufficiently homogeneous. Unpaired Student's *t*‐test was used for comparisons between two groups and analysis of one‐way or two‐way ANOVA for multiple groups, *p < 0.05, **p < 0.01, ***p < 0.001, and ****p < 0.0001, ns was no significance with *p* > 0.05.

## Author Contributions

C.W. helped with bioinformatics analysis, experiments, data analysis, manuscript writing, and figure preparation. Y.G. helped with study design, experiments, and data acquisition. W.D. helped with study design, writing and editing the manuscript. The three co‐first authors all contributed equally to the design of the study. L.X. helped with conducting experiments. J.Q. helped with acquiring data and bioinformatics analysis. Y.Z. helped with conducting experiments and acquiring data. Y.C. helped with conducting experiments and study design. Z.W. helped with study design and editing the manuscript. Y.W. helped with editing the manuscript. Y.S. helped with editing of the manuscript. D.X. helped with providing reagents, data analysis and editing of the manuscript. X. Gao. helped with providing reagents, acquiring data, data analysis, and editing of the manuscript. M.C. helped with the study design and editing of the manuscript. X. Gu. helped with study design, acquiring data, data analysis, conducting experiments, providing reagents, and writing and editing the manuscript.

## Funding

This study was supported by the National Natural Science Foundation of China (Grant No. 82474175, China to X.Gu.); the Science and Technology Innovation Action Plan of Shanghai Science and Technology Commission (Grant No. 23S11900500, China to X.Gu.); and the Shanghai Sailing Program, Shanghai Rising‐Star Project (Grant No. 24YF2727000, China to W.D.). Youth Fund of National Natural Science Foundation of China (Grant No. 82500855, China to W.D.). Research Startup Funding of Shanghai Fourth People's Hospital Affiliated to Tongji University (Grant No. sykyqd11801, China to X. Gao.).

## Conflicts of Interest

The authors declare no conflicts of interest.

## Supporting information




**Supporting File**: advs74661‐sup‐0001‐SuppMat.docx.

## Data Availability

The data supporting the findings of this study are available from the corresponding author upon reasonable request. The single‐cell data presented in this paper are deposited to GSE281245(https://www.ncbi.nlm.nih.gov/geo/query/acc.cgi?acc=GSE281245). Code was deposited to https://github.com/gettygugu‐dot/aa.
