# Supplementary material for: TRAP5 Inhibition Targeting Scar‐Associated Macrophages Ameliorates Acute Kidney Injury to Chronic Kidney Disease Transition
Source: Adv Sci (Weinh). 2026 Mar 3;13(26):e19855. doi: 10.1002/advs.202519855 (PMC13159109; doi:10.1002/advs.202519855)
Supplement: Supplementary file 1 — Supporting File: advs74661‐sup‐0001‐SuppMat.docx. [file ADVS-13-e19855-s001.docx]

Supporting Information

TRAP5 Inhibition Targeting Scar-Associated Macrophages Ameliorates Acute Kidney Injury to Chronic Kidney Disease Transition

Chenxi Wang, Yaodong Gu, Wen Du, Lin Xie, Jinwei Quan, Yu Zhao, Ye Cheng, Zhaonan Wei, Yuanyuan Sha, Yi Wang, Dechao Xu, Xiang Gao*, Min Chen*, Xiangchen Gu

Supplemental Tables

Table S1. Primers sequences

| **Gene**  **(Mouse)** | **Forward** | **Reverse** |
| --- | --- | --- |
| *Fn* | ATGGTACAGCTGATCCTGCC | GCCCTGGTTTGTACCTGCTA |
| *Col3a1* | ACAGCTGGTGAACCTGG | ACCAGGAGATCCATCTCGAC |
| *Col1a1* | CCCAGCCGCAAAGAGTCTAC | AGCATACCTCGGGTTTCCAC |
| *Tgfb1* | GGGAAGCAGTGCCCGAACCC | TGGGGGTCAGCAGCCGGTTA |
| *Nlrp3* | CAAGGCTGCTATCTGGAGGAA | GAGGTCCACATCTTCAAGGTCC |
| *IL-1β* | TGCCACCTTTTGACAGTGATG | TGATGTGCTGCTGCGAGATT |
| *Tnfa* | CCCTCCAGAAAAGACACCATG | CACCCCGAAGTTCAGTAGACAG |
| *Cd68* | AAGGTCCAGGGAGGTTGTGA | GGCTCTGATGTAGGTCCTGT |
| *Cd86* | GATGGACCCCAGATGCACCAT | ACCAGCTCACTCAGGCTTATG |
| *Ccl2* | CTGGAGCATCCACGTGTTGG | TCTTGAGCTTGGTGACAAAAACTAC |
| *Vim* | AGACCAGAGATGGACAGGTGA | TTGCGCTCCTGAAAAACTGC |
| *Gapdh* | AAGAAGGTGGTGAAGCAGGCATC | CGGCATCGAAGGTGGAAGAGTG |
| *Hacvr1* | GCTGCTACTGCTCCTTGTGA | GGAAGGCAACCACGCTTAGA |
| *Acta2* | AGCCATCTTTCATTGGGATGGAG | CTCCTTCTGCATCCTGTCAGC |
| *Acp5* | GCTGTCCTGGCTCAAAAAGC | CACACCGTTCTCGTCCTGAA |
| *Il10* | GCTATGCTGCCTGCTCTTACT | CCTGCTGATCCTCATGCCA |
| *Mrc1* | TCATTCCCTCAGCAAGCGAT | GATACTTGCCAGGTCCCCAC |
| *Mr* | GCCTGGATGTGGTTGGATGTA | AGCCTTTGGTTTCCATAGCTTGA |
| *Ym1* | AAGAGTGCTGATCTCAATGTGGATT | AATTGTAGGGGCACCAATTCC |
| *Spp1* | TGGCTGAATTCTGAGGGACT | TTCTGAGATGGGTCAGGCAC |
| *Mif* | CTTTGTACCGTCCTCCGGTC | GTGCACTGCGATGTACTGTG |
| *Mmp9* | CTCTCCTGGCTTTCGGCTG | GCGGTACAAGTATGCCTCTGC |
| *Ctsk* | GTAGCCACGCTTCCTATCCG | CGAGAGATTTCATCCACCTTGC |
| *Retnla* | ACCTTTCCTGAGATTCTGCCC | CAGTGGAGGGATAGTTAGCTGG |
| *Glud1* | GGACAGGATATCGGGTGCAT | TGTGAAGGTCACACCAGCTT |
| *Gls* | CCGCGGGCGACAATAAAATA | AACCTGGGATCAGATGTTCG |

SUPLEMMENTAL FIGURES


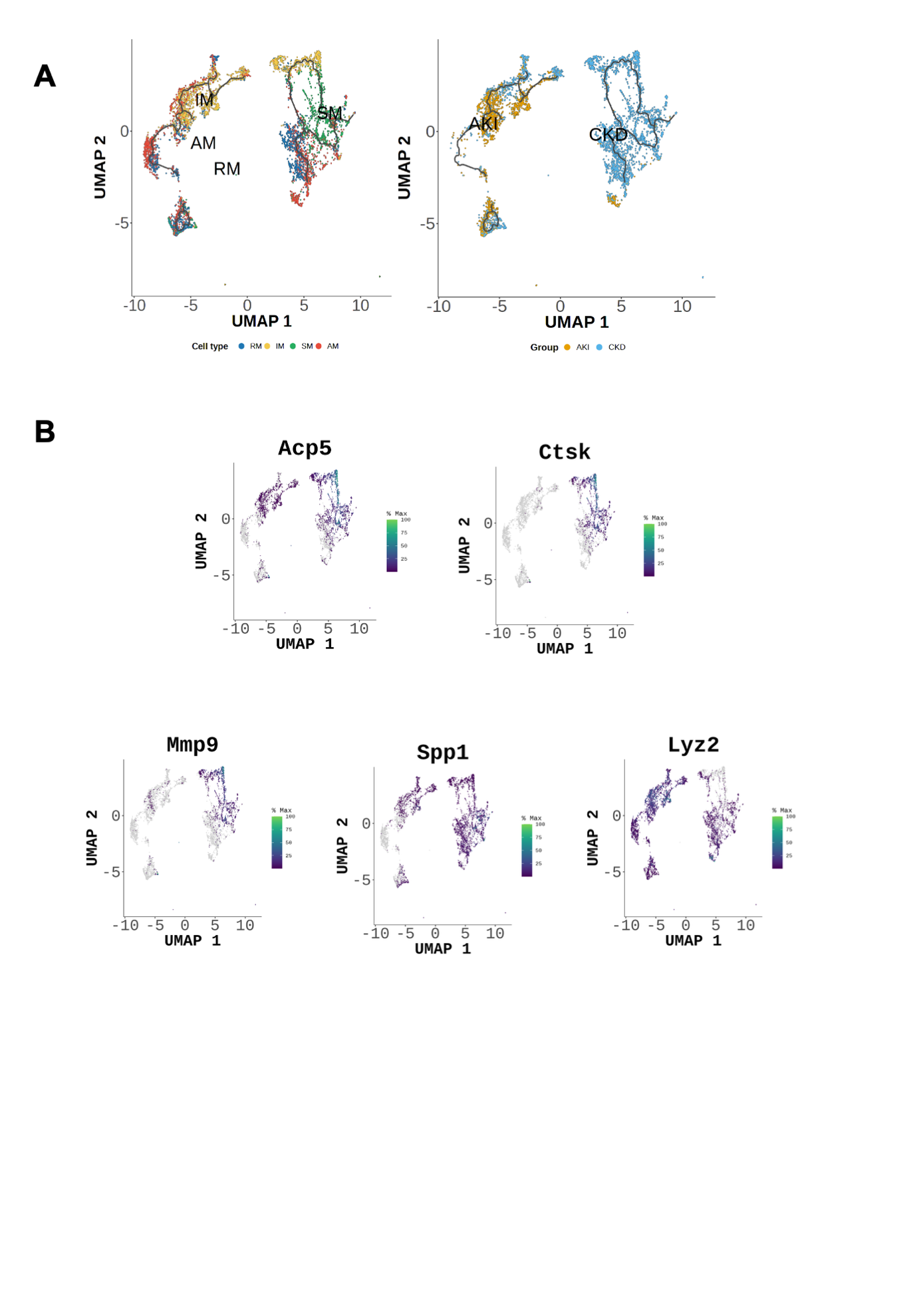


**Figure S1. UMAP of macrophage subclusters and marker genes in AKI and CKD**

(**a**) UMAP embedding of kidney macrophage cells. Left, cells colored by subcluster (RM, IM, SM, AM); right, cells colored by condition (AKI vs. CKD). The black line denotes the Monocle 3 trajectory backbone (learn_graph) inferred from these cells; each dot represents one cell. (**b**) Feature plots showing scaled expression (0–100% of the maximum) of selected genes across the same UMAP: *Acp5*, *Mmp9*, *Ctsk*, *Spp1*, Lyz2. Color intensity reflects normalized expression level.

**
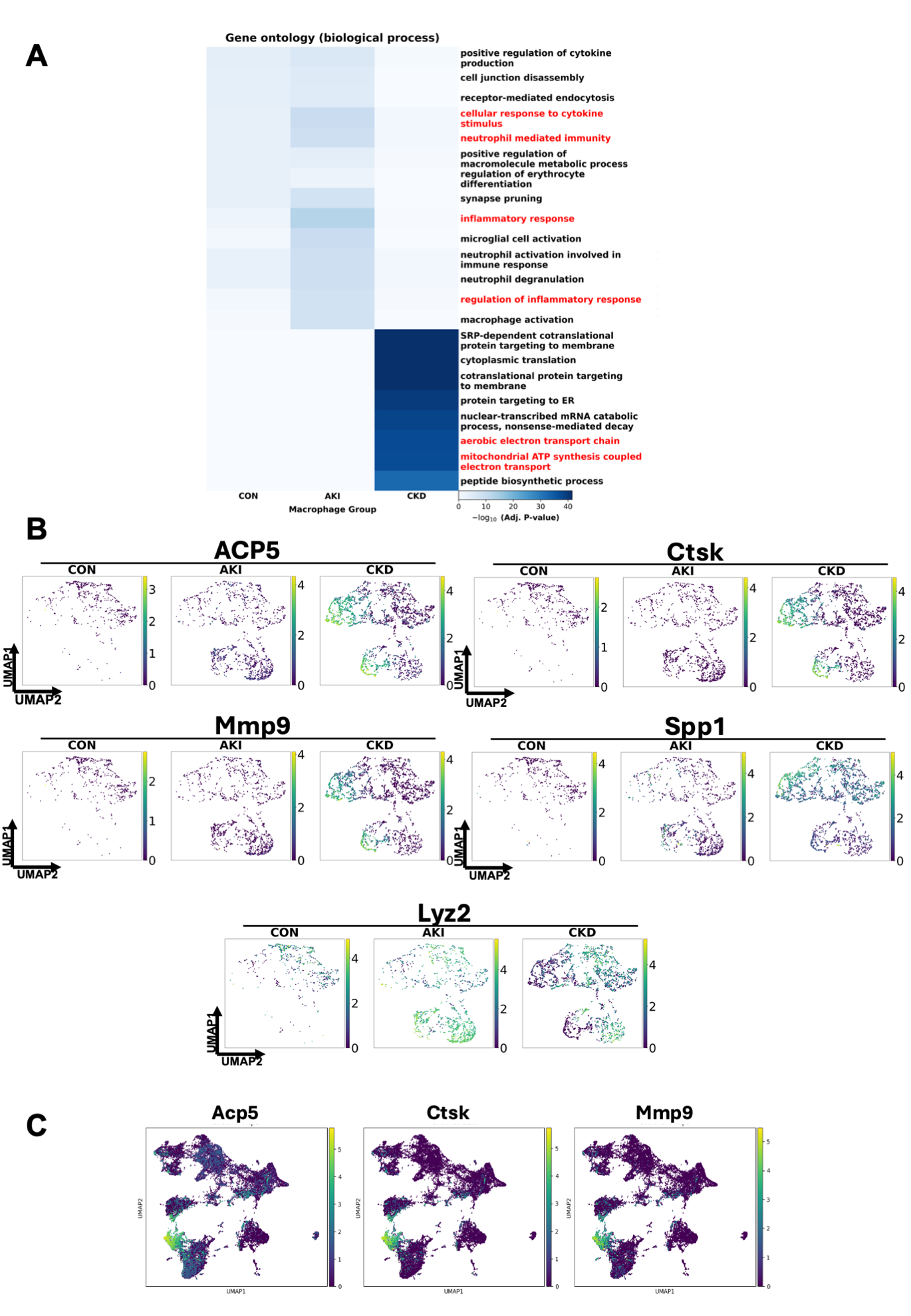
**

**Figure S2. Functional programs and gene expression of kidney macrophages in AKI vs. CKD**

(**a**)Gene Ontology (biological process) enrichment of differentially expressed genes in macrophages from control (CON), AKI, and CKD samples. The heatmap displays enrichment significance as −log10(adjusted P value); darker blue indicates stronger enrichment. Terms highlight inflammatory/immune responses (red labels) in AKI and aerobic electron transport chain and mitochondrial ATP-synthesis coupled electron transport (red labels) in CKD. (**b**)Condition-split UMAP feature plots showing normalized expression of canonical macrophage and activation markers (***Acp5*, *Ctsk*, *Mmp9*, *Spp1*, *Lyz2***) in CON, AKI, and CKD. Each dot is one cell; color intensity reflects relative expression (gray = low/none). (c) UMAP feature plots showing log-normalized expression of *Acp5*, *Mmp9*, and *Ctsk* across the kidney cell atlas.


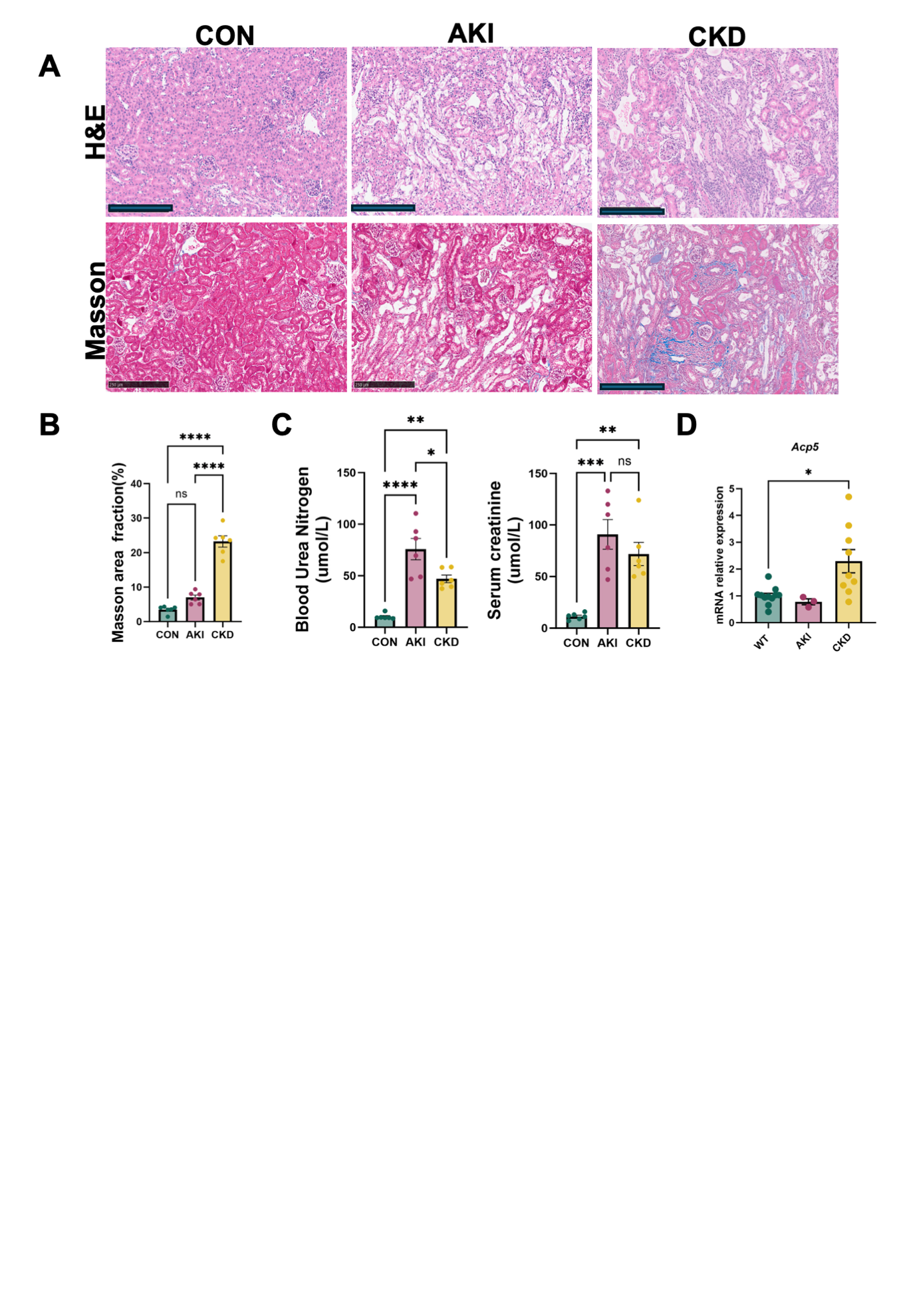


**Figure S3. Kidney histopathology, renal function, and *Acp5* expression in AAI-induced AKI to CKD transition**

**(a)**Representative kidney sections from control (CON), acute kidney injury (AKI), and chronic kidney disease (CKD) groups stained with H&E (top) and Masson’s trichrome (bottom). Scale bars, 250μm. **(b)** Quantification of fibrotic area (Masson-positive fraction) in the three groups. **(c)** Blood urea nitrogen (BUN) and serum creatinine levels. **(d)** Relative *Acp5* mRNA expression by qPCR in kidneys from CON/WT, AKI, and CKD. n=6 per group(**b-c**), n=3-9 in each group(**d**). Data are shown as mean ± SEM. Significance was assessed by One-way ANOVA test with multiple comparisons (ns, not significant; * P<0.05, ****** P <0.01***,* ***** P ***<0.001***, **** P <0.0001).


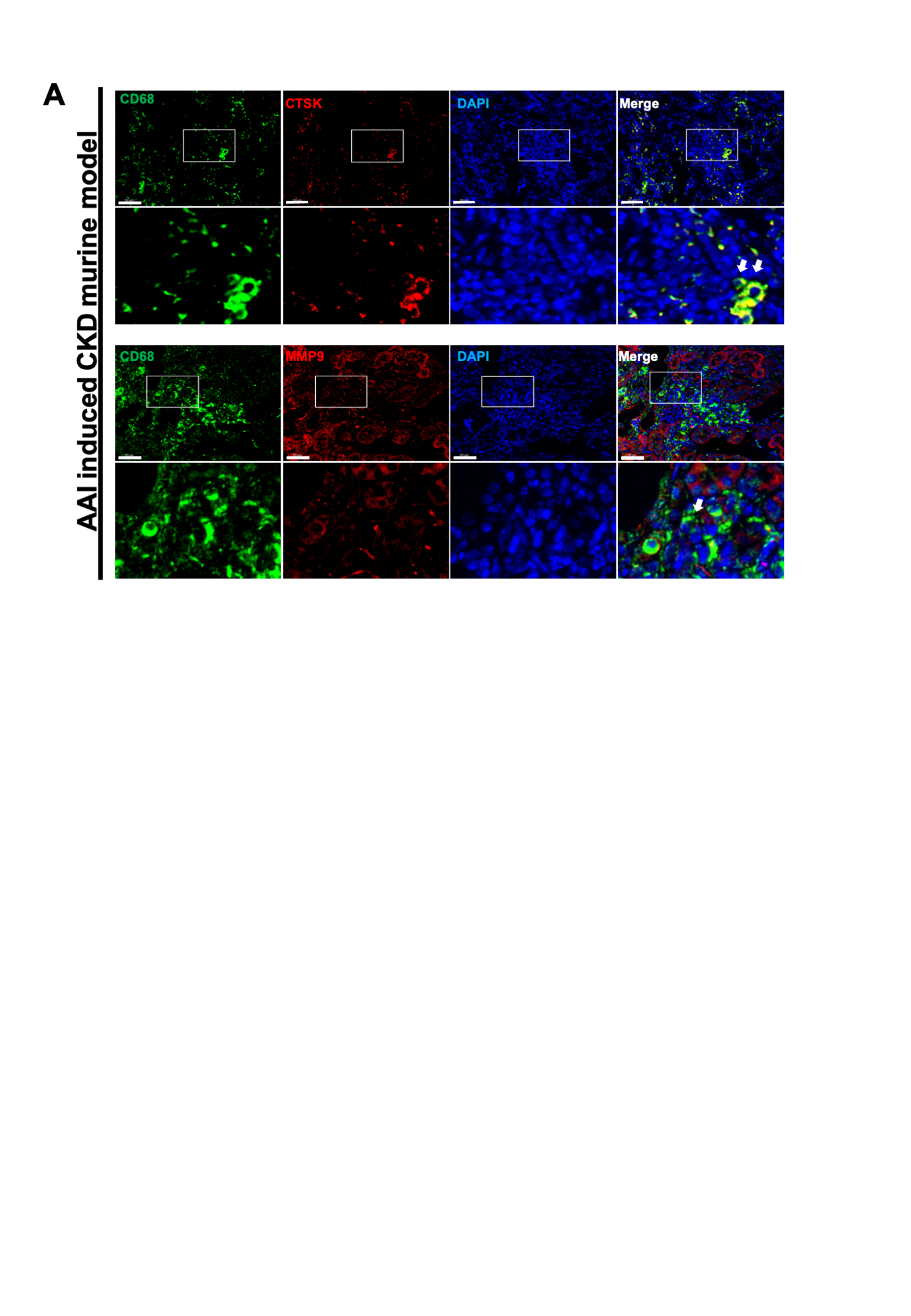
**Figure S4. Macrophage co-localization with CTSK and MMP9 in AAI-induced CKD**

(**a**) Representative immunofluorescence images of kidneys from the AAI-induced CKD mouse model showing macrophages (CD68, green) co-stained with CTSK (red, top row) or MMP9 (red, bottom row); nuclei are counterstained with DAPI (blue). Merged images highlight CD68⁺ cells expressing CTSK or MMP9 (yellow). White boxes indicate regions enlarged in the lower panels; arrows mark examples of co-localized cells. Scale bars, 50 µm.

**
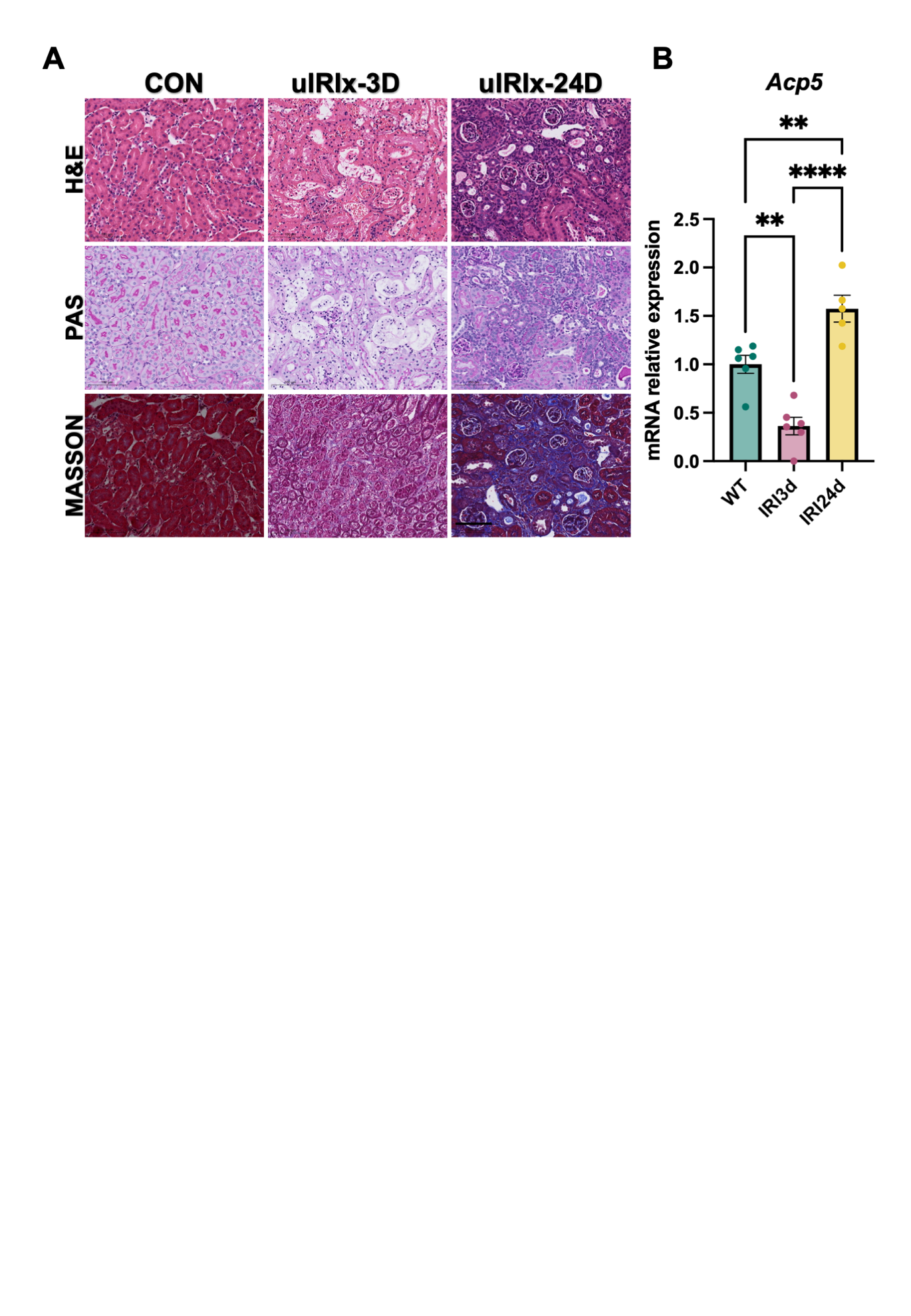
**

**Figure S5. Histopathology and TRAP5/ACP5 expression over time in the uIRIx**

(**a**) Representative kidney sections from control (CON), uIRIx with at day 3 (uIRIx-3D, acute phase) and day 24 (uIRIx-24D, chronic phase). Stains: H&E (top), PAS (middle), and Masson’s trichrome (bottom). uIRIx-3D shows acute tubular injury and brush-border loss, whereas uIRIx-24D exhibits prominent interstitial fibrosis. Scale bars, 100 μm. (**b**) Relative *Acp5* mRNA expression by qPCR in kidneys from CON, uIRIx-3D, and uIRIx-24D groups. n=5-6 per group. Data are shown as mean ± SEM. Significance was assessed by one-way ANOVA with multiple comparisons; ***P* < 0.01, *****P* < 0.0001.

**
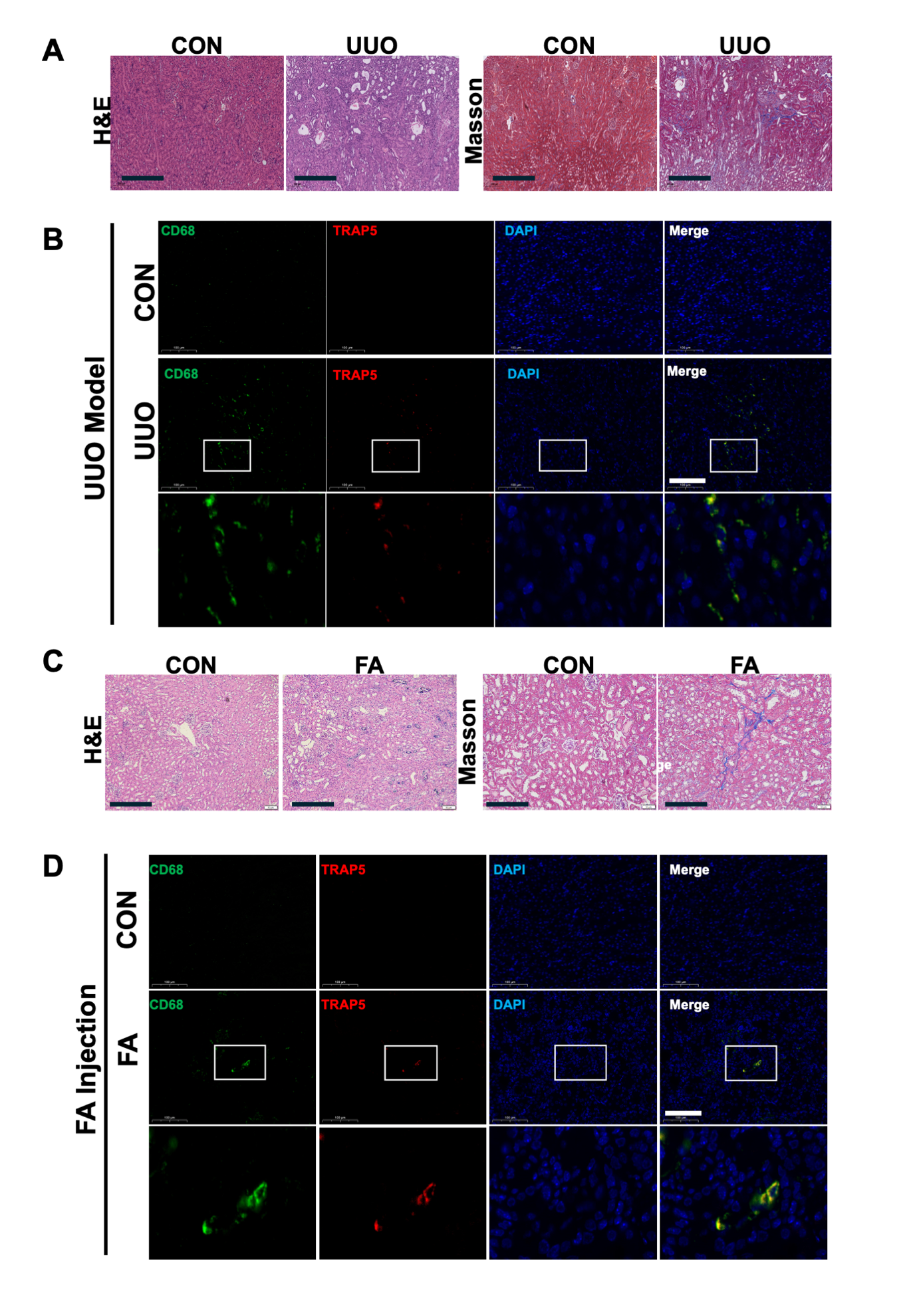
**

**Figure S6. TRAP5⁺ macrophages in UUO and folic-acid induced nephropathy**

(**a**)Representative histology from contralateral (CON) and unilateral ureteral obstruction (UUO) kidneys. H&E (left) shows tubular injury and dilation in UUO; Masson’s trichrome (right) highlights marked interstitial collagen deposition in UUO compared with CON. Scale bars, 100µm. (**b**) Immunofluorescence of UUO kidneys showing macrophages (CD68, green) and TRAP5 (red) with nuclear counterstain (DAPI, blue). Merged images reveal CD68⁺ TRAP5⁺ cells (yellow). Boxes indicate regions enlarged in the bottom panels. Scale bars, 100µm. (**c**) Representative histology from folic-acid (FA)–induced kidney injury versus CON. H&E demonstrates tubular injuries; Masson’s trichrome shows increased interstitial fibrosis after FA. Scale bars, 50µm. (**d**) Immunofluorescence of FA kidneys stained for CD68 (green), TRAP5 (red), and DAPI (blue). Co-localization (yellow) indicates TRAP5 expression in macrophages. Boxed areas are shown enlarged below. Scale bar, 100µm.

**
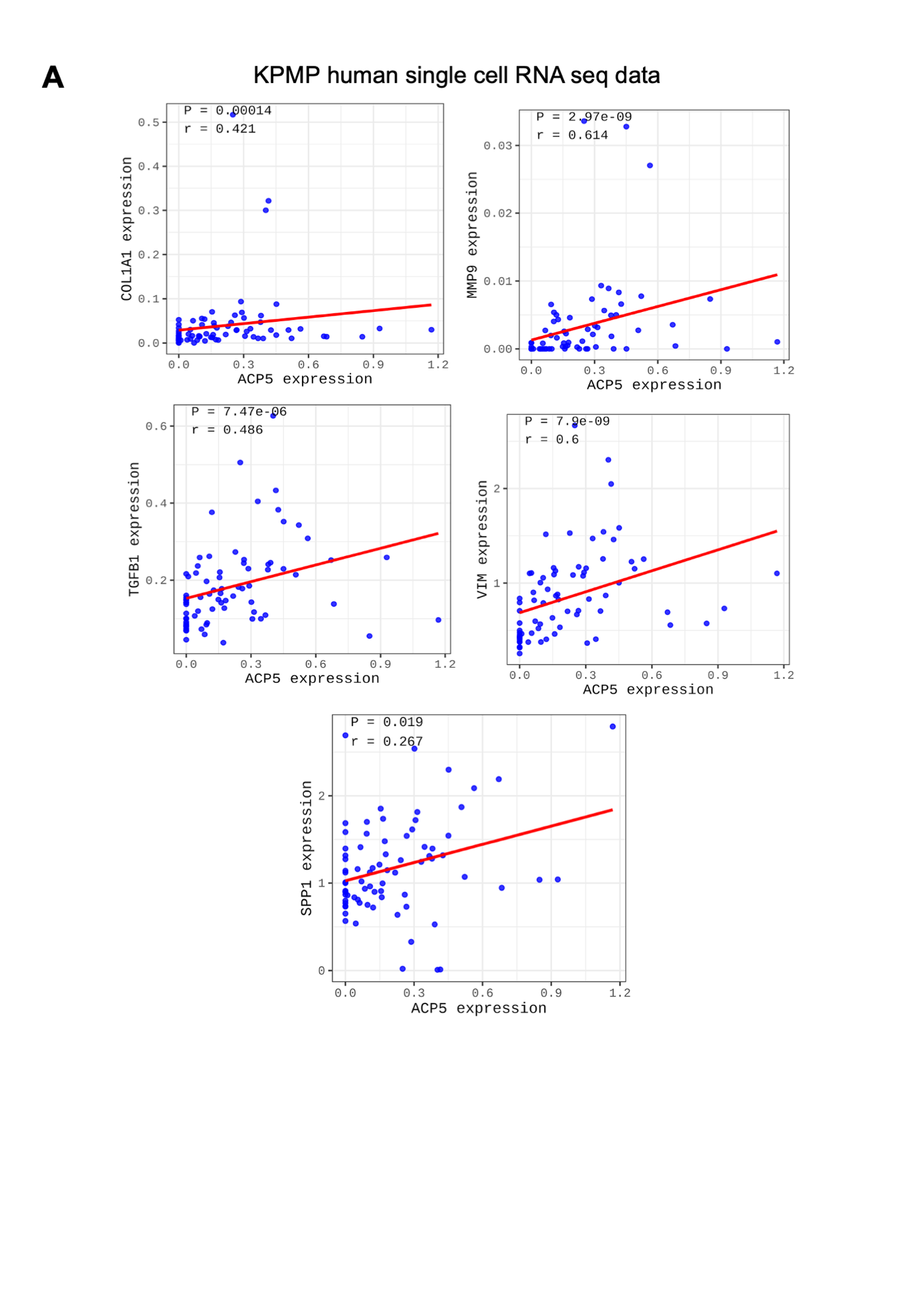
**

**Figure S7 Correlation between macrophage ACP5 expression and fibrotic/injury markers in human kidney single-cell RNA-seq data (KPMP)**

(a) Scatter plots showing sample-level relationships between macrophage *ACP5* expression (x-axis) and expression of *COL1A1*, *MMP9*, *TGFB1*, *VIM*, and *SPP1* (y-axis) in the KPMP human kidney single-cell RNA-seq dataset, which includes control, AKI and CKD samples. For each kidney, mean ACP5 expression was calculated from annotated macrophage clusters, and mean expression of the indicated genes was calculated across all cells. Each dot represents one kidney sample; red lines indicate linear regression fits. Spearman correlation coefficients (r) and P values are shown in each panel.

**
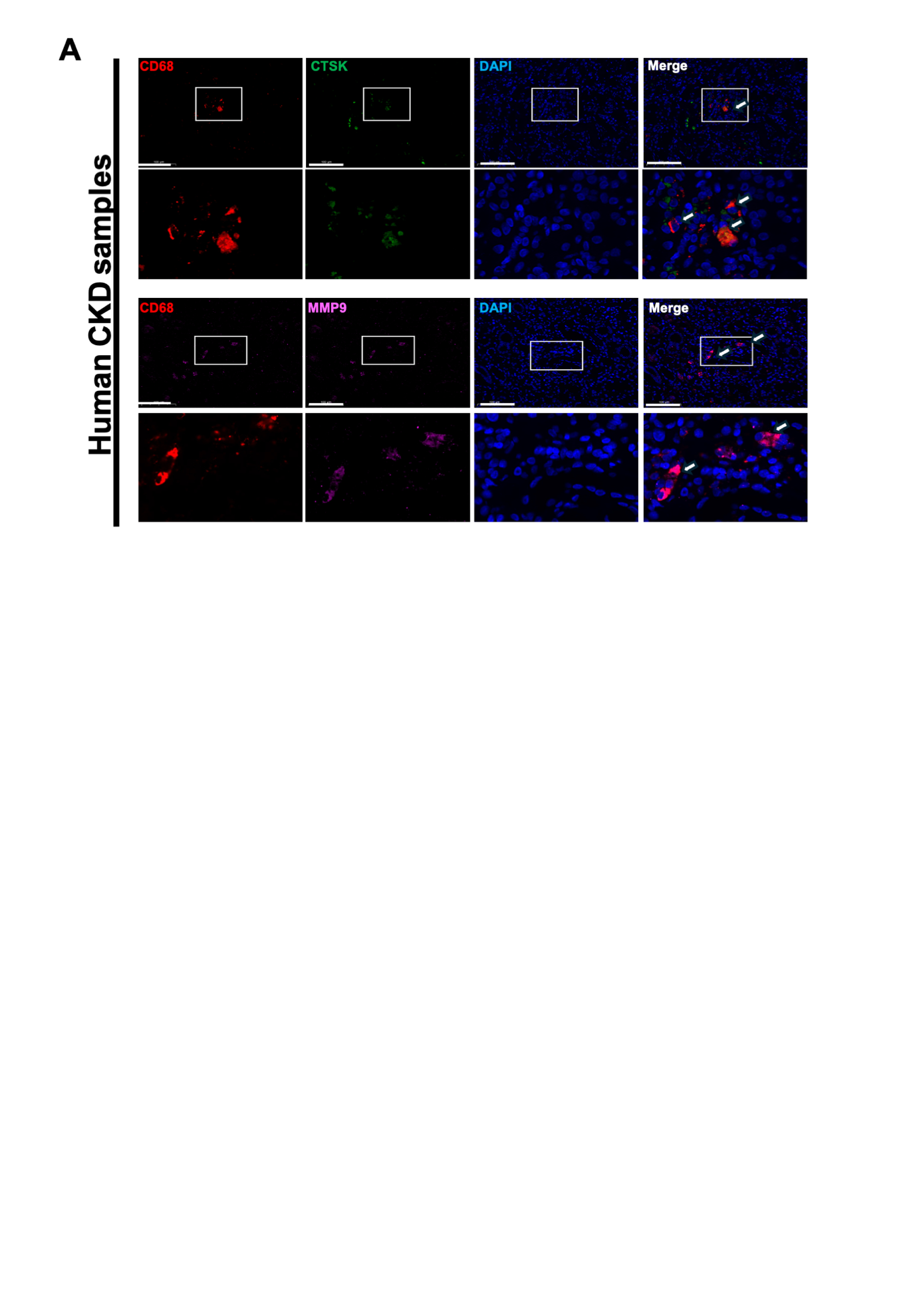
**

**Figure S8. CTSK and MMP9 co-localize with CD68⁺ macrophages in human CKD kidneys**

(**a**)Representative immunofluorescence images from human CKD renal biopsies. Upper row: CD68 (red), CTSK (green), and DAPI (blue); lower row: CD68 (red), MMP9 (magenta), and DAPI (blue). Merged images show CD68⁺ macrophages co-expressing CTSK (yellow overlap) or MMP9 (magenta overlap). White boxes indicate regions enlarged below; arrows highlight double-positive cells. Scale bars, 100 µm.

**
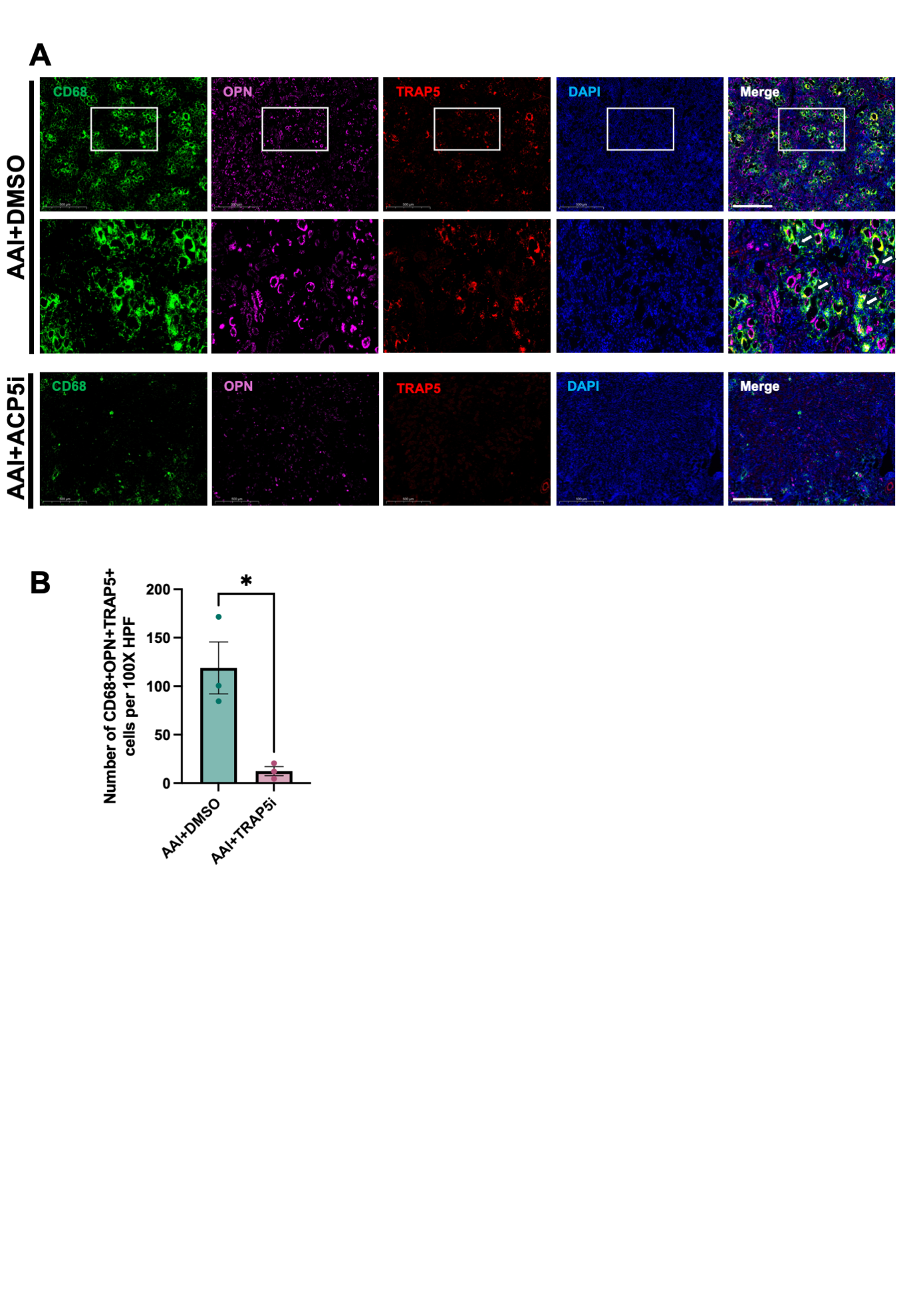
**

**Figure S9. TRAP5 inhibition reduces OPN⁺TRAP5⁺ macrophages in AAI-induced AKI–to–CKD kidneys**

(**a**)Representative immunofluorescence staining for CD68 (green), OPN (magenta), TRAP5 (red) and nuclei (DAPI, blue) in kidneys from AAI-treated mice receiving vehicle (AAI + DMSO, top two rows) or TRAP5 inhibitor (AAI + TRAP5i, bottom row). In the AAI + DMSO group, CD68⁺ macrophages in the interstitium show strong OPN and TRAP5 signals, with the merged image highlighting clusters of OPN⁺TRAP5⁺ CD68⁺ scar-associated macrophages (enlarged views in the second row). In contrast, AAI + TRAP5i kidneys display markedly reduced OPN and TRAP5 staining within CD68⁺ cells, consistent with disruption of the OPN–TRAP5 axis by TRAP5 inhibition. Scale bars, 500 µm. (**b**) Quantification of CD68⁺OPN⁺TRAP5⁺ cells per 100× high-power field (HPF) in kidneys from AAI + DMSO and AAI + TRAP5i groups (n = 3 mice per group). Each dot represents one mouse; bars indicate mean ± SEM. Statistical significance was assessed by unpaired t-test (*P* < 0.05).

**
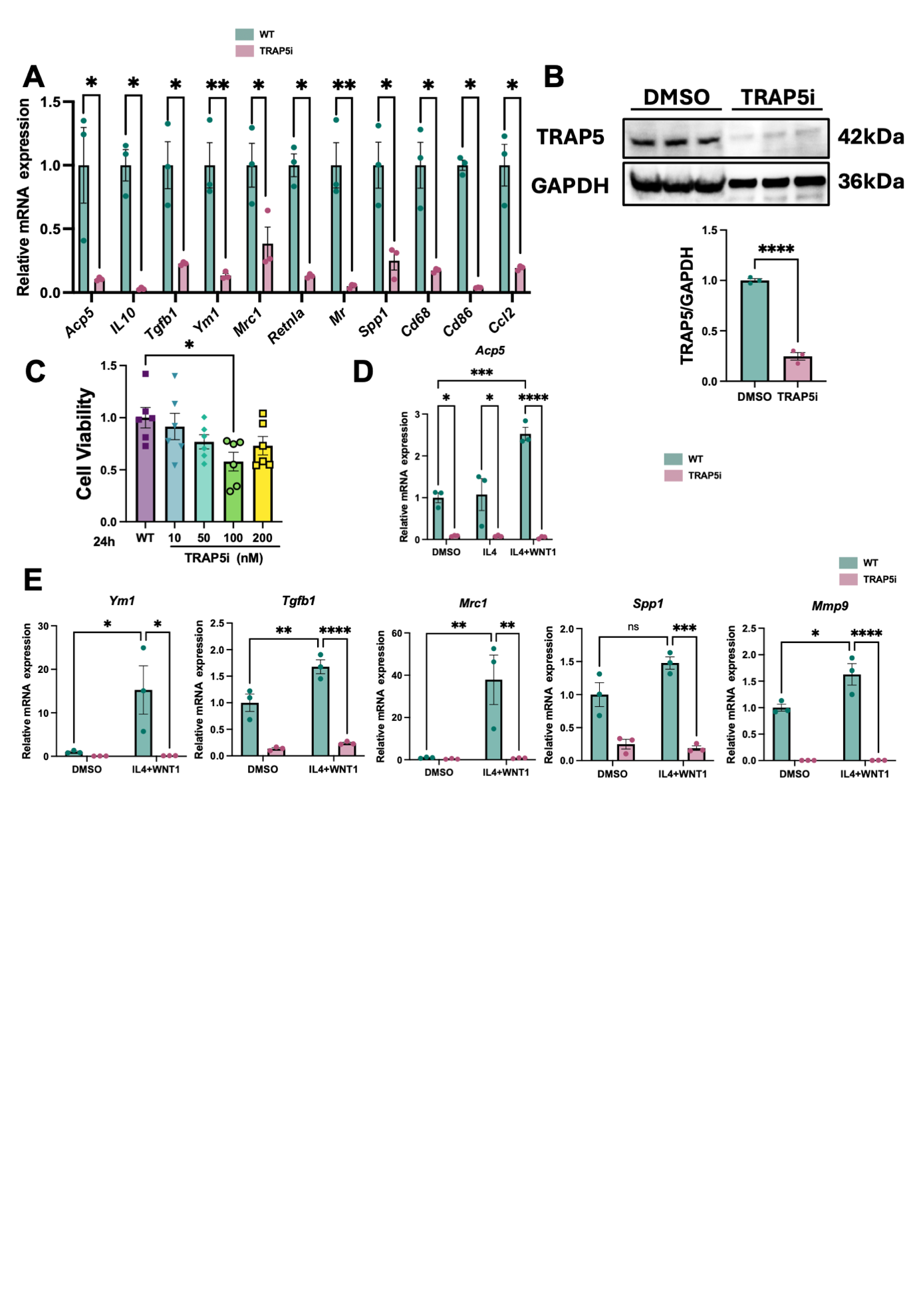
Figure S10. TRAP5 activity sustains IL-4/WNT1–driven alternative macrophage activation**

(**a**)qPCR for *Acp5* and polarization genes (*Il10*, *Tgfb1*, *Ym1*, *Mrc1*, *Retnla*, *Mr*, *Spp1*, *Cd68*, *Cd86*, *Ccl2*) in BMDMs stimulated with TRAP5 inhibitor (TRAP5i) for 24 h (n=3 independent experiments per group). (**b**) Immunoblot for TRAP5 in BMDMs treated as in (**a**), with densitometric quantification normalized to GAPDH showing reduced TRAP5 protein after TRAP5i (n=3 independent experiments per group). (**c**) Cell viability after 24h exposure to TRAP5i in a dose dependent manner (n=6 independent experiments per group). (**d**) *Acp5* mRNA induction by IL-4 alone versus IL-4 + WNT1, relative to DMSO control (n=3 independent experiments per group). (**e**) qPCR confirming that IL-4 + WNT1 upregulates M2 polarized genes (*Ym1*, *Tgfb1*, *Mrc1*, *Spp1*, *Mmp9*) and TRAP5i attenuates these responses(n=3 independent experiments per group). Data are presented as mean ± SEM. Group comparisons in (**a**) and (**b**) were analyzed by unpaired two-tailed Student’s t-test; (**c**) by one-way ANOVA; and (**d**) and (**e**) by two-way ANOVA, each followed by appropriate multiple-comparisons testing (**P* < 0.05, ***P* < 0.01, ****P* < 0.001, *****P* < 0.0001).
